# Exploring the Mechanism of Action of Danggui Buxue Decoction in Treating Chronic Glomerulonephritis Based on UPLC‐MS/MS and Network Pharmacology

**DOI:** 10.1002/iid3.70497

**Published:** 2026-09-11

**Authors:** Qiao Liu, Yinghang Wang, Xinyue Wang, Hongmei Yang, Zhi Pan, Di Guo

**Affiliations:** ^1^ Fangzheng Research Laboratory, Jilin Ginseng Academy Changchun University of Chinese Medicine Changchun Jilin China; ^2^ College of Traditional Chinese Medicine Changchun University of Chinese Medicine Changchun Jilin China; ^3^ Public Experimental Center Changchun University of Chinese Medicine Changchun Jilin China; ^4^ College of Basic Medicine Changchun University of Chinese Medicine Changchun Jilin China

**Keywords:** CGN, DBD, HBZY‐1, network pharmacology, UPLC‐MS/MS

## Abstract

**Ethnopharmacological Relevance:**

According to Chinese medicine theory, chronic glomerulonephritis (CGN) is characterized by dampness‐heat and blood stasis. Danggui Buxue Decoction (DBD) has shown good efficacy in the treatment of diseases associated with qi deficiency and blood stasis (such as CGN). However, the molecular mechanisms behind its therapeutic effects are unclear.

**Aim of the Study:**

This study aims to elucidate the role and mechanism of action of DBD in the treatment of CGN.

**Materials and Methods:**

The chemical composition of DBD was identified using UPLC‐MS/MS, and network pharmacology, bioinformatics, and molecular‐docking techniques were employed to predict the core targets and key signaling pathways of DBD in the treatment of CGN. At the same time, we established an LPS‐induced rat glomerular mesangial (HBZY‐1) cell model and a CGN rat model for in vitro and in vivo validation. We used MTT and Transwell assays to assess the viability, migration, and invasion capabilities of HBZY‐1 cells, and also measured inflammatory cytokine levels via ELISA. A preliminary evaluation of the regulatory effects of DBD on key proteins in the TLR4/NF‐κB signaling pathway in HBZY‐1 cells using immunofluorescence. Biochemical analyses of urine and serum were performed to measure 24‐h urinary protein, Serum Creatinine (SCr), Blood Urea Nitrogen (BUN), and levels of inflammatory factors in rats. Pathological changes in kidney tissue were examined using HE, Masson, and PAS staining. The extent of apoptosis in kidney tissue cells was assessed using TUNEL and JC‐1 staining assays. Immunohistochemistry, immunofluorescence, RT‐qPCR, and Western blotting were used to detect the expression of relevant mRNAs and proteins in kidney tissue.

**Results:**

The UPLC‐MS/MS method was used to identify 104 chemical components and 499 potential targets of action in DBD. The 166 potential target genes in DBD are associated with the therapeutic mechanism of CGN. Results from network pharmacology and bioinformatics suggest that the NF‐κB signaling pathway may be a potential mechanism underlying the treatment of CGN with DBD. In vitro experiments demonstrated that DBD suppressed the survival rate of HBZY‐1 cells, upregulated the secretion of Interleukin‐ 4 (IL‐4) and Interleukin‐ 10 (IL‐10), and downregulated the expression of Toll‐Like Receptor 4 (TLR4) and Nuclear Factor kappa‐B (NF‐κB) proteins. In vivo, DBD downregulated 24‐h urinary protein, SCr, BUN, Tumor Necrosis Factor‐α (TNF‐α), and Interleukin‐ 6 (IL‐6) levels in CGN rats, while upregulating IL‐4 and IL‐10 levels. At the same time, DBD exerts its therapeutic effect on CGN by inducing apoptosis through a reduction in mitochondrial membrane potential and by alleviating the inflammatory response via inhibition of the TLR4/NF‐κB pathway.

**Conclusions:**

In summary, DBD may exert a therapeutic effect on CGN by regulating the TLR4/NF‐κB signaling pathway, thereby suppressing the inflammatory response and modulating apoptosis.

## Graphical abstract

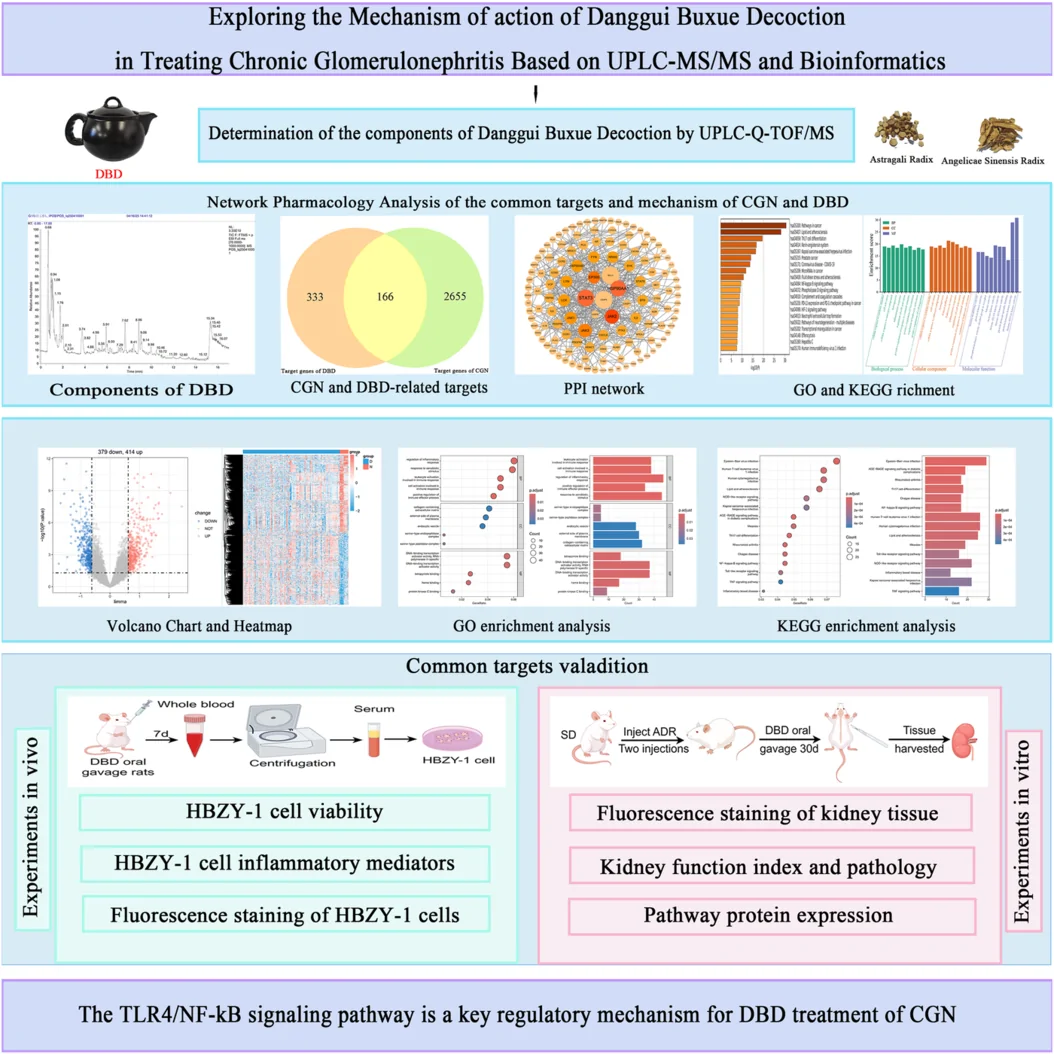

**Abbreviations**
AR: astmgali radix
ASR: angelicae sinensis radix
Bax: Bcl‐2‐Associated X Protein
Bcl‐2: B‐cell lymphoma‐2
BUN: blood urea nitrogen
Caspase‐3: cysteine‐dependent aspartate‐specific protease‐3
Caspase‐9: cysteinyl aspartate specific proteinase‐9
CGN: chronic glomerulonephritis
Cyt C: cytochrome C
DBD: Danggui Buxue Decoction
GEO: gene expression omnibus
GO: gene ontology
HBZY‐1: rat glomerular mesangial cells
IL‐10: interleukin‐ 10
IL‐4: interleukin‐ 4
IL‐6: interleukin‐ 6
IκBa: nuclear factor of kappa light polypeptide gene enhancer in B‐cells inhibitor
KEGG: kyoto encyclopedia of genes and genomes
MyD88: myeloid differentiation primary response 88
NFκB: nuclear factor‐κappaB
PPI: protein–protein interaction
SCr: serum creatinine
TLR4: toll‐like receptor 4
TNF‐α: tumor necrosis factor‐α

## 1 Introduction

Chronic glomerulonephritis (CGN) is a relatively common type of chronic kidney disease, typically causing glomerular injury and tubular dysfunction, and progressively advancing to kidney fibrosis [1]. In recent years, there has been an increasing number of people with CGN, and it's really threatening to our health [2]. This disease causes progressive damage to kidney function, leads to a variety of serious complications, significantly reduces patients’ quality of life, and increases the burden of disease. Although we don't know exactly what causes CGN, studies have demonstrated that there is a critical part for apoptosis in the start and development of the disease [3, 4].

Apoptosis, as type I cell death, is an active death process strictly controlled by multiple genes [5]. This process is essential for maintaining glomerular cell homeostasis and processing damaged cells [6]. It has been shown that in the pathological process of CGN, the abnormal behavior of the rat glomerular mesangial cells (HBZY‐1) is a central aspect that drives the disease progression [7]. On the one hand, inhibition of apoptosis leads to pathological accumulation of HBZY‐1, promoting inflammatory responses and extracellular matrix deposition [8]. On the other hand, excessive activation of apoptosis accelerates the loss of glomerular intrinsic cells and disrupts filtration function. This imbalance in apoptosis is closely associated with clinical manifestations of CGN, such as Serum Creatinine (SCr), Blood Urea Nitrogen (BUN), and proteinuria, suggesting that targeted regulation of apoptosis may represent a novel therapeutic strategy for CGN.

Among the many classic formulas of traditional Chinese medicine, Danggui Buxue Decoction (DBD) occupies an important position as a traditional medicine for tonifying qi and blood [9]. DBD was first documented in the Jin‐Yuan period in Li Dongyuan's “Neiwaishang Bianhuo Lun”, about 800 years ago. Specifically, DBD is composed of two herbs, Angelicae Sinensis Radix (ASR) and Astmgali Radix (AR), in a ratio of 1:5 [10]. Use of both together could produce a combined immunoregulatory, hemodynamic, anti‐inflammatory, and antioxidant effect by the complementary synergy of the major ingredients. Furthermore, relevant research has demonstrated that DBD can cause cell death through the upregulation of pro‐apoptosis proteins and thus slow down the development of colorectal cancer [11]. Furthermore, it demonstrates that DBD induces bone marrow cell proliferation and apoptosis and improves the microcirculation of bone marrow [12]. DBD can also directly act on cardiomyocytes, protecting the heart by influencing mitochondrial apoptotic changes [13]. Existing research indicates that DBD exerts kidney protective effects through multiple mechanisms. DBD could improve diabetic nephropathy by regulating the carbonyl compound metabolic profile, AGEs/RAGE pathway [14]. At the same time, DBD could also slow down the kidney fibrosis process by regulating the autophagy through miR ‐ 27a/PI3K/AKT pathway [15]. Additionally, studies have found that DBD activates the Notch signaling pathway, promoting wound healing in rat kidneys [14]. Our group's prior research has demonstrated that DBD alleviates kidney injury by reducing apoptosis in interstitial fibrosis cells and inhibiting epithelial‐mesenchymal transition [16]. However, the key components of DBD therapy for CGN, the associated targets, and their mechanisms of action remain poorly understood. Therefore, in this study, we employed UPLC‐MS/MS technology to systematically identify the active components of DBD. By integrating network pharmacology and bioinformatics approaches, we predicted the core targets and potential mechanisms of action for CGN. Subsequent in vitro and in vivo experiments validated the aforementioned hypothesis and provided an in‐depth explanation of its regulatory mechanisms.

## 2 Methods

### 2.1 Materials and Reagents

For more details, please refer to the Supporting Information Table S1.

### 2.2 The Preparation of DBD

ASR and AR were purchased from Jilin Province Bei‐Yao Traditional Chinese Medicine Pharmaceutical Group Co. Ltd. (China). Both herbs were immersed in distilled water for 30 min, boiled for 45 min, and filtered to collect the primary filtrate. Next, distilled water was added to the herbal residue and simmered for another 45 min. Then filter, collect the two filtrates, and concentrate them to the respective DBD concentrations (Table 1). Refrigerate at 4°C until ready to use.

**Table 1:** Composition of DBD decoction.

Chinese name	English name	Latin name	genus	Part used	Dose (g)
Danggui	Angelica	Angelica sinensis (Oliv.) Diels	Apiaceae	Root	5
Huangqi	Astragalus	Astragalus membranaceus (Fisch.) Bge	Legume	Root	25

### 2.3 UPLC‐MS/MS Analysis

LC/MS data collection for DBD was performed using a Vanquish ultra‐high‐performance liquid chromatograph (Thermo, USA) and a QExactive HFX mass spectrometer (Thermo, USA). At a column temperature of 40°C, the sample solution was separated on a Waters HSS T3 column (100 mm × 2.1 mm, 1.8 μm). Mobile phase A: 0.1% formic acid in water; B: 0.1% formic acid in acetonitrile; flow rate: 0.3 mL/min. Elution gradient: 0 min Phase A/Phase B (100:0, v/v), 1 min Phase A/Phase B (100:0, v/v), 12 min Phase A/Phase B (5:95, v/v), 13 min Phase A/Phase B (5:95, v/v), 13.1 min Phase A/Phase B (100:0, v/v), 17 min Phase A/Phase B (100:0, v/v). Equipped with an electrospray ionization source, 40 arb of sheath gas, 10 arb of carrier gas, and a positive/negative ion scanning mode.

### 2.4 Network Pharmacology Analysis

Based on the UPLC‐MS/MS results, the structures of the bioactive compounds were obtained from the PubChem (https://pubchem.ncbi.nlm.nih.gov) and SwissTargetPrediction (http://www.swisstargetprediction.ch) databases, and their targets were predicted. Search for disease‐related targets in the GeneCards (https://www.genecards.org/) and OMIM (https://www.omim.org/) databases using the keyword “CGN”. Using the UniProt database (https://www.uniprot.org/), drug and disease targets were standardized to their official gene names. The intersection of these two sets was then calculated using the Venny 2.1.0 (https://bioinfogp.cnb.csic.es) online tool to identify potential targets for DBD treatment. Import the relevant data using Cytoscape 3.7.0 software to construct a “Traditional Chinese Medicine – Constituents – Diseases – Targets” network diagram.

The common targets of drugs and diseases were uploaded to the STRING database (https://cn.string-db.org/) to construct a protein–protein interaction (PPI) network using Cytoscape 3.7.0. The top 10 core targets in the PPI network were ranked using CytoHubba (MCC algorithm) and the MCODE plugin. Subsequently, a enrich­ment analysis was performed using the Metascape online analysis platform.

### 2.5 Bioinformatics Analysis

This study integrated gene expression data sets from the Gene Expression Omnibus (GEO) (https://www.ncbi.nlm.nih.gov/geo/) database (GSE116626: 74 CGN samples and 7 controls samples; GSE104066: 70 CGN samples and 6 controls samples), which were standardized and batch‐corrected using the “sva” package in R. Differential expression analysis of CGN and control samples was performed using the limma package, and differentially expressed genes (DEGs) were identified using a threshold of |log2 FC| > 1 and a corrected *p*‐value < 0.05. Additionally, we used the pheatmap and ggplot2 packages to create volcano plots and heatmaps to visualize the DEGs.

Subsequently, the clusterProfiler package was used to perform Gene Ontology (GO) functional annotation, Kyoto Encyclopedia of Genes and Genomes (KEGG) pathway enrichment analysis, and gene set enrichment analysis (GSEA) on the DEGs, with a corrected *p*‐value < 0.05 serving as the criterion for determining statistical significance of the enrichment results.

### 2.6 Molecular Docking

Sort the active components of DBD by their “degree” values and select those with a “degree” value of 10 or higher for molecular docking with key proteins. Three‐dimensional structure of the target protein from the protein data bank (https://www.rcsb.org/), and the structure of the small molecule ligands from the PubChem database (https://pubchem.ncbi.nlm.nih.gov/). Calculate the binding energy using AutoDock software and visualize the docking results using PyMOL software.

### 2.7 Preparation of Medicated Serum

Male SD rats (180–200 g) were purchased from Changchun Yisi Laboratory Animal Technology Co. Ltd. All procedures involving animals were approved by the Animal Ethics Committee of Changchun University of Traditional Chinese Medicine and complied with the National Standards for Laboratory Animals. According to the body surface area conversion factors for humans and rats outlined in Experimental Methods in Pharmacology, the oral dose for rats is 10 mL/kg, which is 6.3 times the equivalent dose for humans (body surface area conversion factor). 30 SD rats were randomly divided into 5 groups (*n* = 6): the Control group (administered an equal volume of saline via gavage), the prednisolone acetate group (PAT: 4.5 mg/kg), and the DBD‐L, DBD‐M, and DBD‐H groups (1.89 g/kg, 3.78 g/kg, 7.56 g/kg). A random number generator was used to form groups to ensure unbiased allocation, and the experiment was conducted as a single‐blind study to minimize observer bias. Finally, blood was drawn from the abdominal aorta, and the serum was separated and set aside.

### 2.8 CGN Model Preparation and Animal Administration

After 7 days of acclimatization feeding, all rats were randomly divided into 6 groups (*n* = 10), including Control, CGN, PAT, DBD‐L, DBD‐M, and DBD‐H. A random number generator was used to form groups to ensure unbiased allocation, and the experiment was conducted as a single‐blind study to minimize observer bias. All rat groups except the control were subjected to CGN model induction via two tail vein injections of ADR [17]. 3.5 mg/kg ADR was injected on the 1st day and 3.0 mg/kg ADR was injected on the 14th day and control was injected with saline at the same times. On day 21, collect 24‐h urine samples from each group of rats. If the 24‐h urinary protein content exceeds 50 mg/kg, the model establishment is considered successful. One week after the successful establishment of the CGN model, the CON group and the CGN group were intragastrically administered an equal volume of normal saline daily. Each treatment group was simultaneously given the corresponding concentration of DBD, and the intervention period for each group was a continuous 30 days. After a month, the urine was collected again. Once sampling is complete, draw blood from the abdominal aorta to isolate serum, and preserve the kidneys.

### 2.9 HBZY‐1 Cell Culture and Cell Administration

HBZY‐1 cells were purchased from Shanghai Fuheng Biotechnology Co. Ltd (Shanghai) and cultured in DMEM medium containing 10% FBS at 37°C. Cells were divided into the following groups: Control group (blank serum), LPS group (LPS + blank serum), PAT group (LPS + 10% PAT drug‐containing serum), DBD‐L group (LPS + 10% DBD‐L drug‐containing serum), DBD‐M group (LPS + 10% DBD‐M drug‐containing serum), and DBD‐H group (LPS + 10% DBD‐H drug‐containing serum). Except for the Control group, all other groups of HBZY‐1 cells were treated with 0.5 μg/mL LPS for 24 h, followed by treatment with the corresponding drug‐containing serum for an additional 24 h [18].

### 2.10 Cell Viability Determination

HBZY‐1 cells (3 × 10^4^cells/well) were seeded onto 96‐well plates. Then add MTT, 20 μL/well, after treatment for 4 h. Add 150 μL DMSO to each well and then measure OD values at a wavelength of 490 nm.

### 2.11 ELISA Assay

Collect cell culture supernatants and blood samples from each group, centrifuge them, and collect the supernatant. Tumor Necrosis Factor‐α (TNF‐α), Interleukin‐6 (IL‐6), Interleukin‐4 (IL‐4), and Interleukin‐10 (IL‐10) levels were measured according to the ELISA kit instructions.

### 2.12 Transwell Experiments

To assess the cells' migratory capacity, 3 × 10^5^ HBZY‐1 cells were seeded into the upper chamber of a Transwell device, and the lower chamber was filled with FM medium containing 10% FBS to induce migration. After 24 h, remove the remaining cells from the upper chamber. The cells that had migrated to the lower chamber were fixed with polyformaldehyde for 30 min and stained with crystal violet for 10 min. For cell invasion assays, use Transwell plates coated with Matrigel; all other steps are the same as for the migration assay. Finally, the cells are observed under a microscope and counted.

### 2.13 Biochemical Analysis

After allowing urine and blood samples to stand, centrifuge them to separate the serum, and measure the concentrations of 24‐h urine protein, SCr, and BUN.

### 2.14 Histological Staining

After the kidney tissue was fixed with 4% paraformaldehyde for 24 h, gradient ethanol dehydration, xylene transparency, paraffin impregnation, and embedding were carried out successively. The paraffin was then sectioned into 5‐μm‐thick sections, which were baked, deparaffinized, and rehydrated before being stained with HE, Masson, and PAS stains and examined under a microscope.

### 2.15 Immunohistochemical Staining

Paraffin sections of kidney tissue were taken, deparaffinized, hydrated, and subjected to antigen extraction with citrate buffer. After treating the tissue sections with 3% hydrogen peroxide and blocking with goat serum, they were incubated overnight at 4°C with the primary antibody for Cleaved Cysteine‐aspartic protease‐3 (Cleaved Caspase‐3) and Toll‐Like Receptor 4 (TLR4), followed by incubation with the corresponding secondary antibody. Afterward, hematoxylin restaining, dehydration, clearing, sealing, and photography were conducted.

### 2.16 Immunofluorescence Staining

After the HBZY‐1 cells were fixed, washed with PBS, permeabilized with 0.5% Triton X‐100, and blocked with 5% BSA, the cells were incubated with TLR4 and Nuclear Factor‐κappaB (NF‐κB) primary antibodies for 24 h. After blocking the kidney tissue sections with 5% goat serum for 30 min, they were incubated with TUNEL, NF‐κB, and Myeloid Differentiation Primary Response 88 (MyD88) primary antibodies for 24 h. The next day, after washing the PBS, add the corresponding secondary antibody and incubate for 1 h. Finally, after 5 min of DAPI staining, examine the sections under a laser confocal microscope.

### 2.17 Mitochondrial Membrane Potential

Fresh kidney tissues were minced to prepare a suspension, followed by centrifugation at 4°C. Then extract the mitochondria and add JC‐1 staining solution, incubate at 37°C for 20 min, and observe the cells under a fluorescence microscope. Finally, the intensity ratio of the two fluorophores was determined using ImageJ software.

### 2.18 RT‐qPCR Assay

Isolation of total RNA from kidney tissue. cDNA was synthesized from RNA via reverse transcription and used as a template for RT‐qPCR with β‐actin as the internal control. Finally, the ratio of the genes of interest is calculated based on the relative Ct value using 2^−ΔΔCt^. RT‐qPCR Amplification Primer Sequences (Table 2).

**Table 2:** Primers for qRT‐PCR.

Gene symbol	Primer sequence (5′ → 3′)
*TLR4*	Forward sequence: *TGTATCGGTGGT* Reverse sequence: *CTGGGGAGGCACA*
*IκBα*	Forward sequence: *TGCACTTGGCCATCATCCAT* Reverse sequence: *TCTGTTGACATCAGCCCCAC*
*NF‐κB*	Forward sequence: *GCATTCTGACCTTGCCTATC* Reverse sequence: *CCAGTCTCCGAGTGAAGC*
*β‐actin*	Forward sequence: *GTCGTACCACTGGCATTGTG* Reverse sequence: *TCTCAGCTGTGGTGGTGAAG*

### 2.19 Western Blotting Assay

The kidney tissue was lysed on ice, and then the protein concentration was determined using the BCA method. Ten micrograms of total protein were subjected to 10% SDS‐PAGE electrophoresis and transferred to a PVDF membrane; the samples were incubated with the primary antibody overnight and with the secondary antibody for 1 h. After development, the gray values were analyzed.

### 2.20 Statistical Analysis

All experiments were performed with at least three independent biological replicates. Numerical data were assessed for normality using standard normality testing procedures. All data are expressed as mean ± SEM. For data following a normal distribution, one‐way analysis of variance (ANOVA) was performed, followed by Tukey's post hoc test. Bonferroni correction was additionally applied to all pairwise comparisons between groups. All data analyses were performed using GraphPad Prism software. When *p* < 0.05, it means the difference between groups is statistically significant.

## 3 Results

### 3.1 Qualitative Analysis of DBD

**Figure 1:**
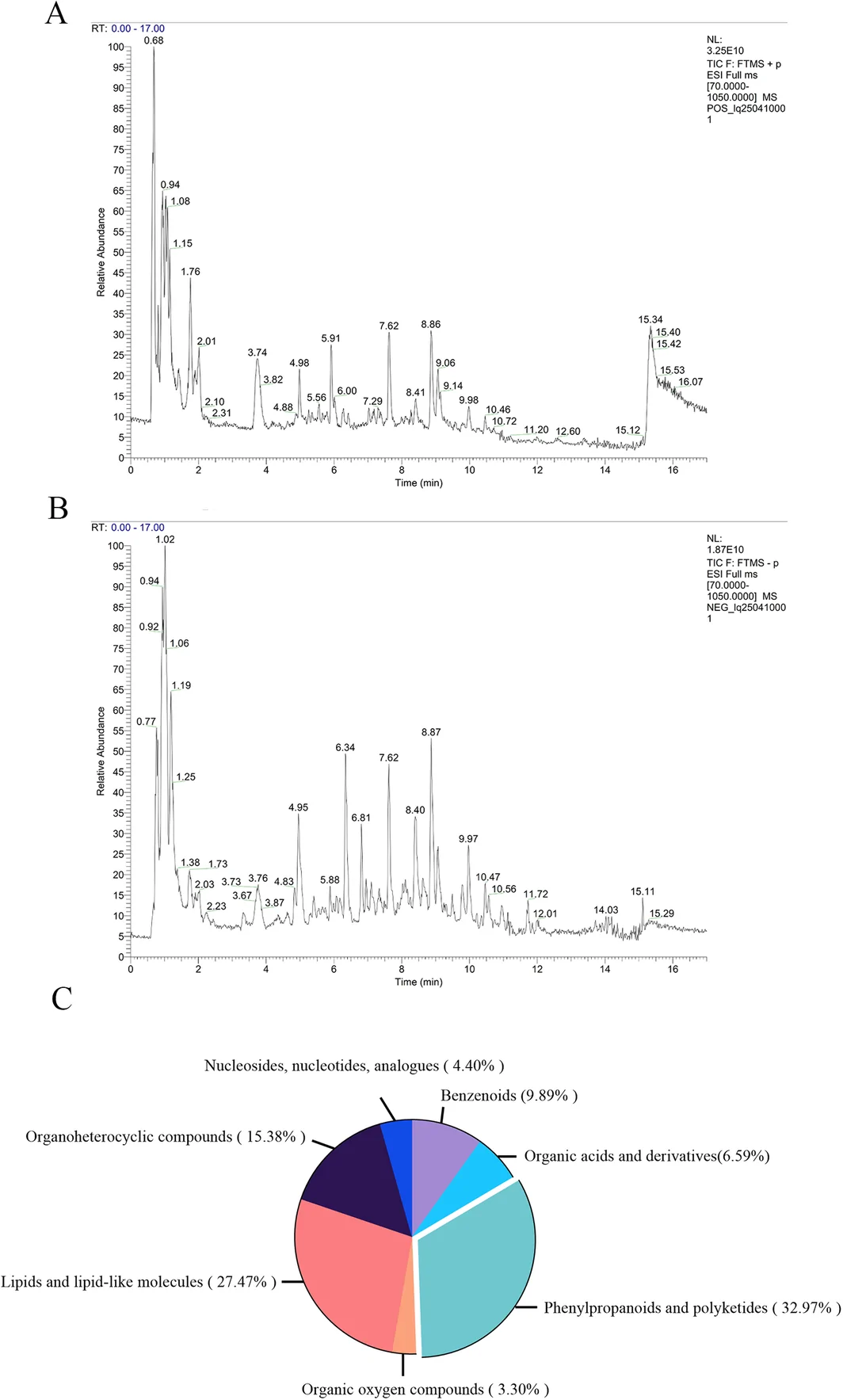
Chromatogram of DBD in UPLC‐MS/MS. (A) Negative spectra of DBD; (B) Positive spectra of DBD; (C) Pie chart showing the breakdown of the compound.

UPLC‐MS/MS was used to perform qualitative analysis of DBD, yielding both positive and negative ion chromatograms (Figure 1A,B). A total of 104 compounds were identified, see Supporting Table S2 for details. The results show that these 104 compounds mainly include effective components such as Phenylpropanoids and polyketides, Lipids and lipid‐like molecules, as well as Organoheterocyclic compounds. DBD compounds were categorized using ClassyFire and displayed in a pie chart (Figure 1C).

### 3.2 Network Construction and Pathway Analyses

**Figure 2:**
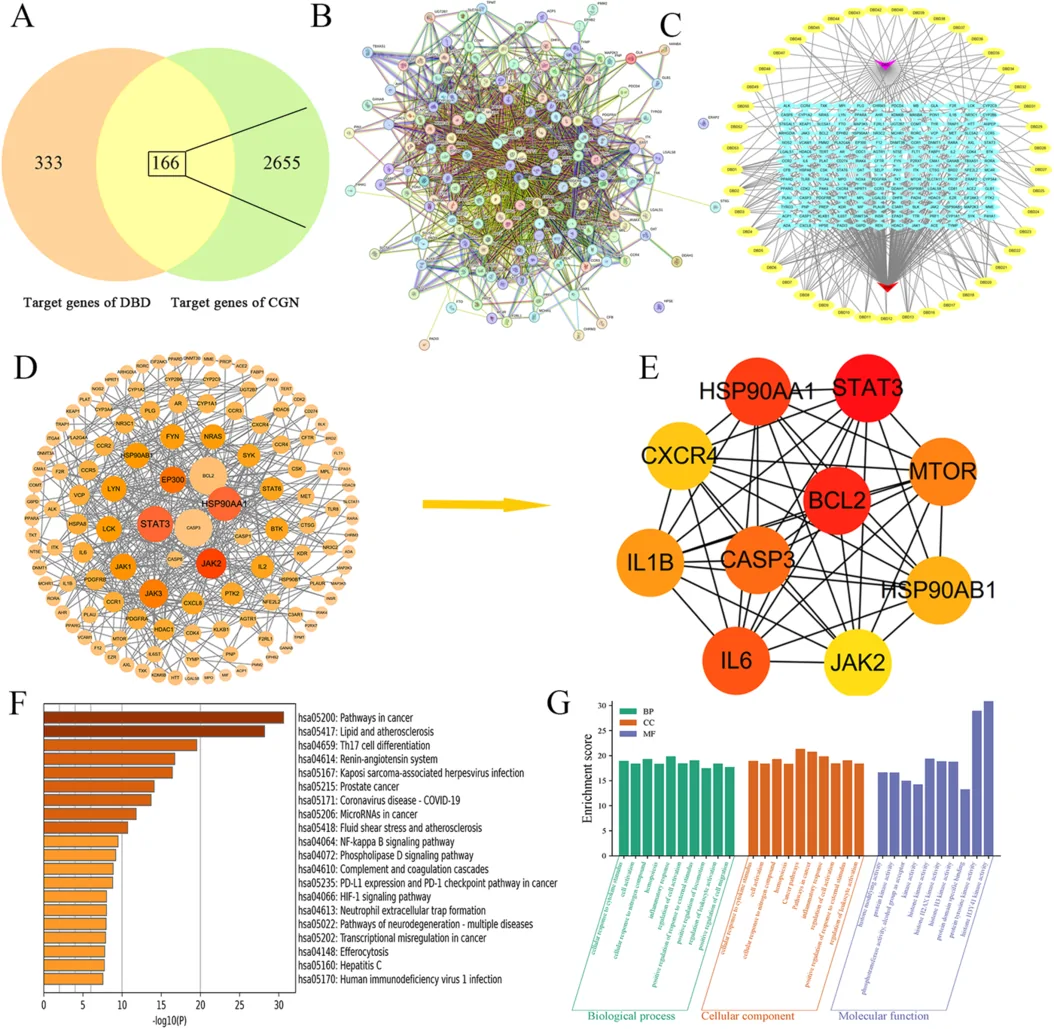
Network Pharmacology Analysis Results. (A) 166 overlapping gene symbols between the CGN and DBD; (B) PPI network analysis; (C) Chinese Medicine‐Composition‐Disease‐Target; (D) Visualizing PPI Networks; (E) Top Ten Core Targets; (F) KEGG enrichment analysis; (G) GO enrichment analysis.

Based on the analysis results of UCPLC‐MS/MS, we used the SwissTargetPrediction database to predict the targets of the obtained active ingredients, and a total of 499 DBD‐related targets were obtained. After screening and redundancy removal, 2821 disease targets were obtained from the GeneCards and OMIM databases. There were a total of 166 cross‐targets between the two (Figure 2A). The PPI network could distinguish the interactions between drug‐related targets and disease targets (Figure 2B). A network diagram of “herbal medicine‐component‐disease‐target” was constructed (Figure 2C). Visualization of the PPI network using Cytoscape (Figure 2D) identified the top 10 key targets in the network, including B‐cell lymphoma‐2 (Bcl‐2) and Cysteine‐dependent aspartate‐specific protease‐3 (Caspase‐3) (Figure 2E).

KEGG enrichment analysis was conducted through the Metascape database, and the results showed that the NF‐κB signaling pathway was significantly involved (Figure 2F). In the BP and CC analyses, the main enrichment is in cellular response to cytokine stimulus, cell activation, and cellular response to nitrogen compounds, etc. In the MF analysis, the main enrichment is to histone modifying activity, protein kinase activity, and phosphotransferase activity, alcohol group as acceptor, etc. (Figure 2G).

### 3.3 Bioinformatics Analysis

**Figure 3:**
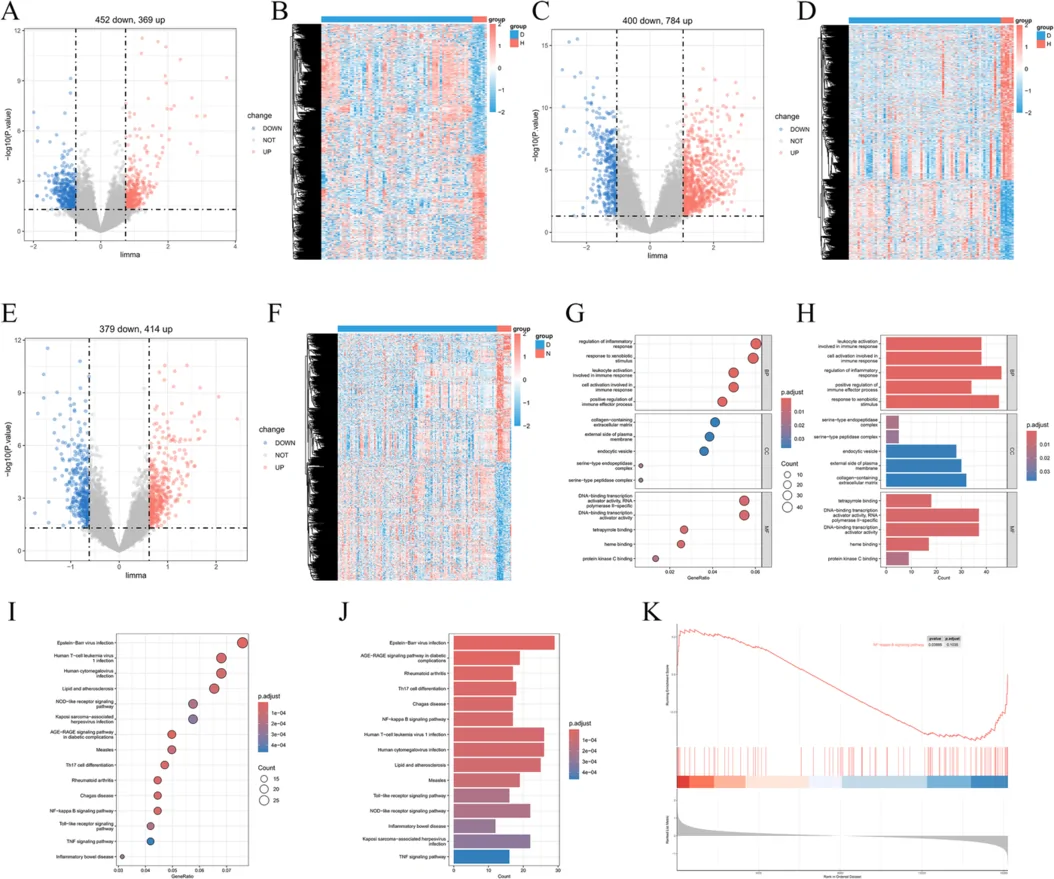
Bioinformatics Analysis. (A–D)Volcano Plot and heatmap of differentially expressed genes in GSE116626, volcano Plot and heatmap of differentially expressed genes in GSE104066; (E, F) Merge GSE116626 and GSE104066 Data sets: Volcano Plot and Heatmap; (G, H) The bubble chart and bar chart display the GO analysis; (I, J) The bubble chart and bar chart display the KEGG analysis; (K) GSEA enrichment analysis curve chart data sets.

There are significant differences in gene expression patterns between healthy individuals and CGN patients. Using R software, generate volcano plots and heatmaps of differentially expressed genes for the GSE116626 and GSE104066 data sets, respectively (Figure 3A–D). The two data sets were then batch‐corrected and standardized, resulting in a single combined dataset. Subsequently, comprehensive data generated volcano plots and heatmaps, identifying 414 significantly upregulated genes and 379 significantly downregulated genes (Figure 3E,F). GO analysis results indicate that regulation of inflammatory processes and response to xenobiotic stimulus are the primary BP. In CC, DEGs are primarily distributed in collagen‐containing extracellular matrix and the external side of the plasma membrane. In MF, DNA‐binding transcription activator activity, RNA polymeraseⅡ‐specific, and DNA‐binding transcription activator activity functions are significantly enriched (Figure 3G). KEGG analysis indicates that DEGs are significantly enriched in the NF‐κB signaling pathway (Figure 3H). At the same time, the findings of this study confirm the strong association between CGN and the NF‐κB signaling pathway in network pharmacology (Figure 3I).

### 3.4 Molecular Docking

**Figure 4:**
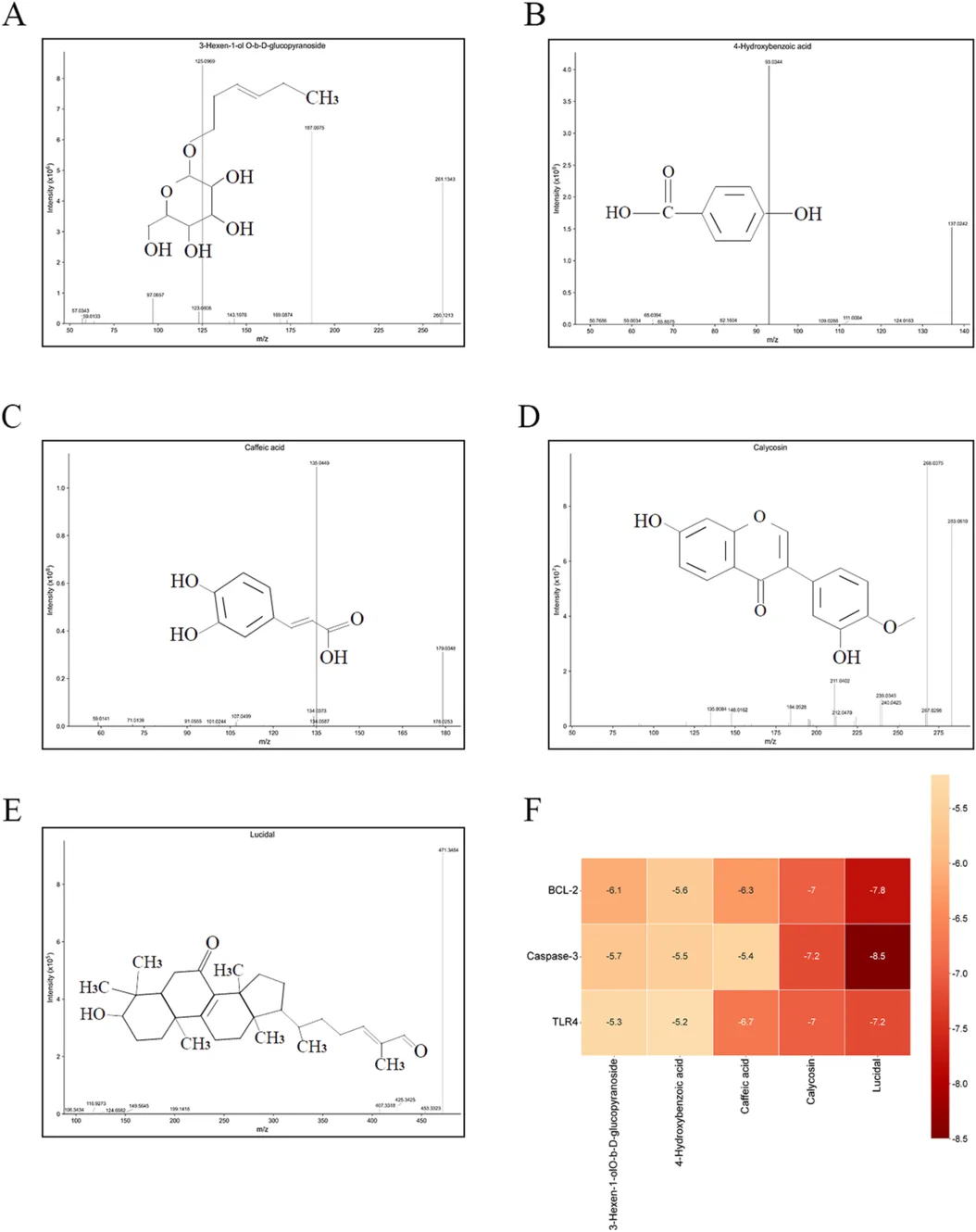
Chemical Structure. (A–E) The chemical structures of formononetin and calycosin; (F) Docking results.

**Figure 5:**
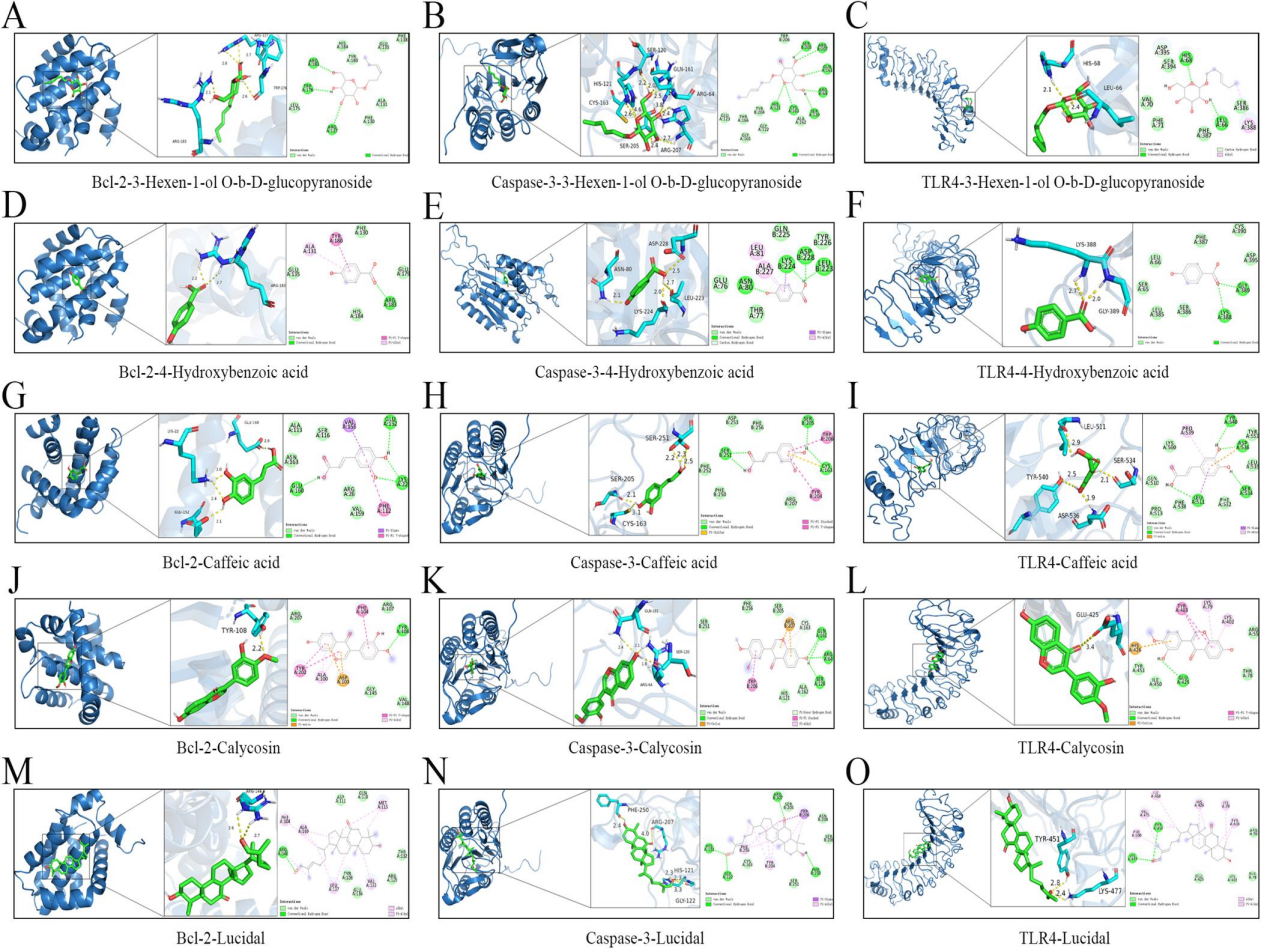
Molecular docking result. (A–O) Schematic diagram of the docking of the key active ingredient with the target protein.

Binding affinities below and includes −5 kcal/mol are generally considered strong binding interactions, and the lower the value more binding(Y [15].). Based on the aforementioned research methodology, five active ingredients with a degree value of 10 or higher were ultimately identified and designated as the key active ingredients: 3‐Hexen‐1‐ol O‐b‐d‐glucopyranoside, 4‐Hydroxybenzoic acid, Caffeic acid, Calycosin, Lucidal. The chemical structures of the key active ingredients are shown in Figure 4A–E. These five active ingredients were subjected to molecular docking with Bcl‐2, Caspase‐3, and TLR4, and all exhibited binding energies of less than −5 kcal/mol (Figure 4F). The binding energy data are shown in Table 3. The results are shown in Figure 5A–O.

**Table 3:** Component‐target molecule docking results.

Compound	CAS	Target	Affinity (kcal/mol)	Number of hydrogen bonds	amino acid residue
3‐Hexen‐1‐olO‐β‐d‐glucopyranoside	95632‐87‐4	Bcl‐2	−6.1	3	ARG127, TRP176, ARG183
		Caspase‐3	−5.7	7	HIS121, CYS163, SER120, ARG6, GLN161, ARG207, SER205
		TLR4	−5.3	2	HIS68, LEU66
4‐Hydroxybenzoic acid	99‐96‐7	Bcl‐2	−5.6	1	ARG183
		Caspase‐3	−5.5	4	ASN80, LYS224, ASP228, LEU223
		TLR4	−5.2	2	LYS388, GLY389
Caffeic acid	331‐39‐5	Bcl‐2	−6.3	3	GLU160, LYS22, GLU152
		Caspase‐3	−5.4	3	SER251, SER205, CYS163
		TLR4	−6.7	4	LEU511, SER534, ASP536, TYR540
Calycosin	20575‐57‐9	Bcl‐2	−7.0	1	TYR108
		Caspase‐3	−7.2	3	SER120, ARG64, GLN161
		TLR4	−7.0	1	GLU425
Lucidal	252351‐96‐5	Bcl‐2	−7.8	1	ARG146
		Caspase‐3	−8.5	4	ARG207, HIS121, GLY122, PHE250
		TLR4	−7.2	2	LYS477, TYR451

### 3.5 Effect of DBD on the Biological Functions of LPS‐Induced HBZY‐1 Cells

During the preparation of the drug‐containing serum, the rats remained in good health, with no adverse reactions or deaths. Blood collection and serum separation were completed according to the scheduled timeline. The resulting samples were pale yellow and clear, with no hemolysis or precipitation. The serum preparation met the required standards and was suitable for subsequent in vitro experiments.

**Figure 6:**
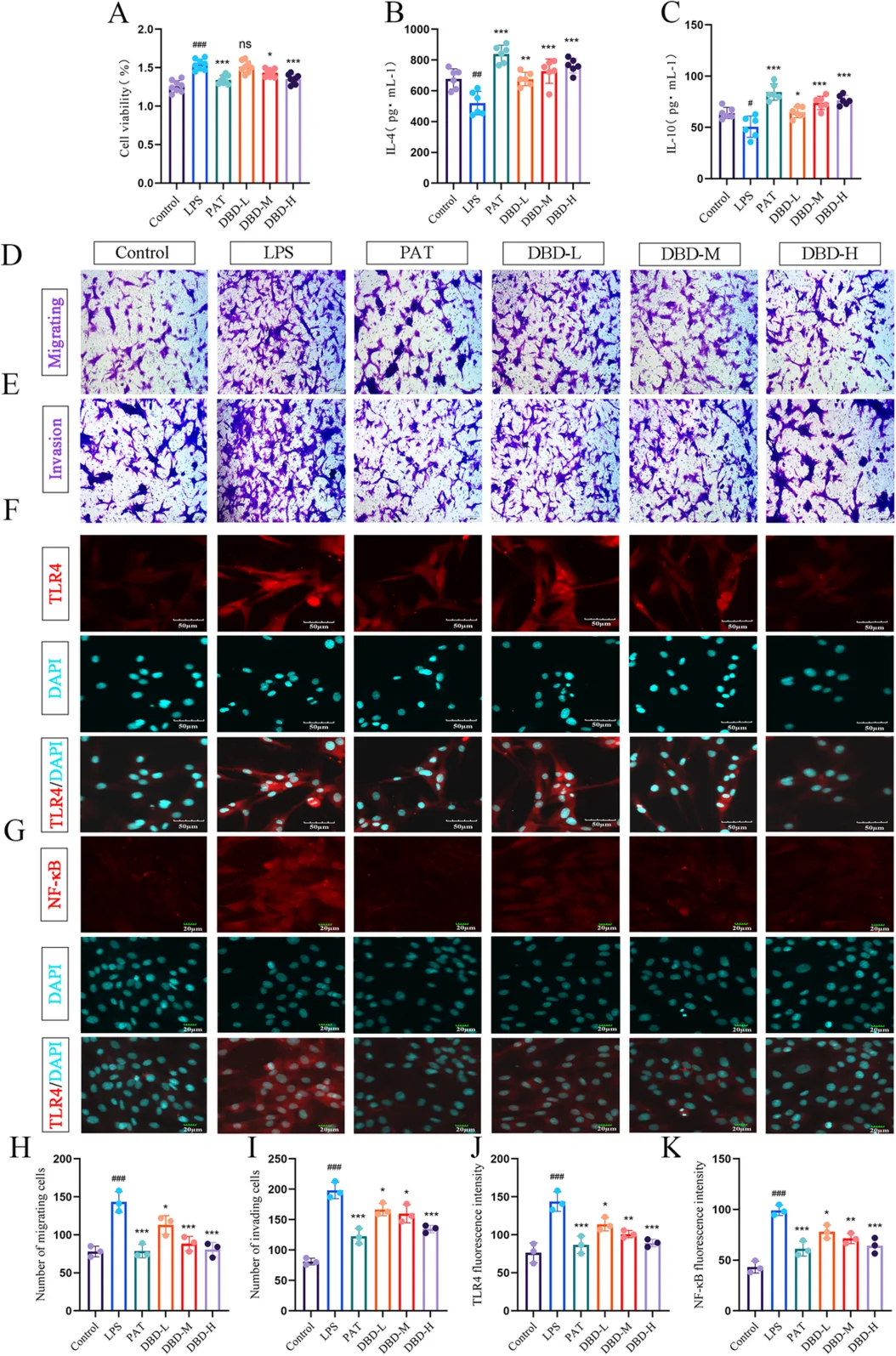
The intervention effect of DBD on HBZY‐1 cells induced by LPS. (A) Viability of each cell group; (B, C) Levels of IL‐4 and IL‐10 secretion in each cell group; (D, E) Representative fluorescence images of migration and invasion in each cell group; (F, G) Representative immunofluorescence images of TLR4 and NF‐κB protein expression in each cell group; (H, I) Quantitative analysis of cell migration and invasion; (J, K) Quantitative analysis of TLR4 and NF‐κB protein expression; Data are expressed as mean ± SD. ^#^ *p* < 0.05, ^##^ *p* < 0.01, ^###^ *p* < 0.001 vs Control; ns indicates no significance; *p < 0.05, **p < 0.01, ***p < 0.001 vs LPS. A, *n* = 8; B–C, *n* = 6; D–K, *n* = 3.

We used MTT and ELISA assays to assess the effects of DBD on the viability of HBZY‐1 cells and the levels of inflammatory cytokines (Figure 6A–C). The results showed that cell viability in the LPS group was significantly increased, while levels of IL‐4 and IL‐10 were significantly decreased. After treatment with PAT and DBD‐containing serum, the aforementioned symptoms improved significantly.

We used a Transwell assay to evaluate the effect of DBD on the migration and invasion capabilities of HBZY‐1 cells (Figure 6D,E). Compared with the Control group, LPS stimulation significantly increased the migration and invasion of HBZY‐1 cells. After treatment with PAT and DBD‐containing serum, HBZY‐1 had much lower migration and invasion capacities than the LPS group.

We evaluated the effects of DBD on the expression of TLR4 and NF‐κB proteins in HBZY‐1 cells using immunofluorescence assays (Figure 6F,G). The fluorescence intensity of TLR4 and NF‐κB proteins in LPS group cells was significantly elevated compared to the Control group. Following PAT and DBD‐containing serum interventions, the fluorescence intensity decreased significantly. The experimental results above indicate that DBD‐containing serum inhibits the proliferation of HBZY‐1 cells and downregulates the expression of TLR4 and NF‐κB proteins.

### 3.6 Effects of DBD on Kidney Function Parameters in Rats With ADR‐Induced CGN

**Figure 7:**
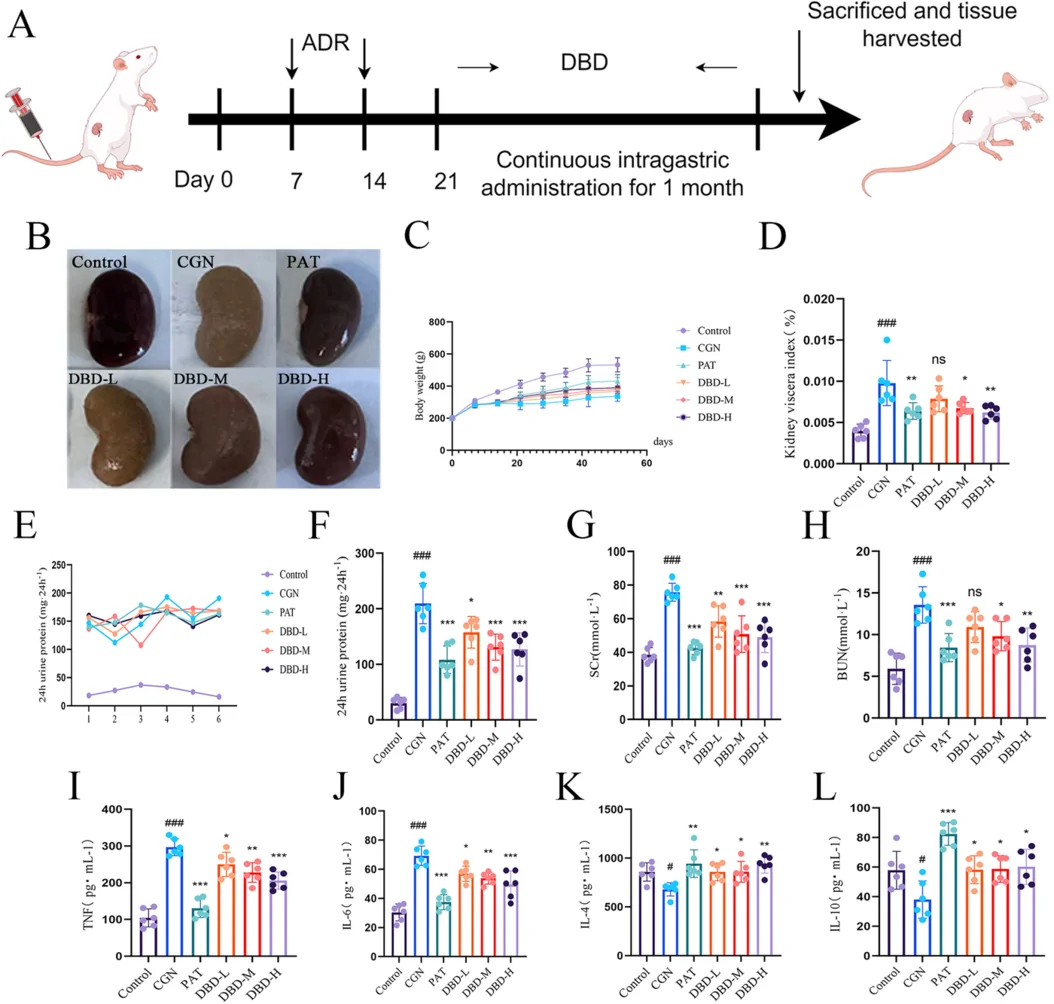
Effects of DBD on kidney function parameters in rats with ADR‐induced CGN. (A) Schematic representation of ADR‐induced CGN rat model and DBD intervention; (B) Representative photographs of morphological changes in the kidneys of different groups; (C) Changes in body weight of rats in each group; (D) Kidney viscera index by group; (E, F) Changes in 24‐h urine protein before and after modeling; (G, H) Changes in SCr,BUN of rats in each group; (I–L) TNF‐α, IL‐1β, IL‐4, and IL‐10 levels in each group; Data are expressed as mean ± SD. ^#^ *p* < 0.05, ^##^ *p* < 0.01, ^###^ *p* < 0.001 vs Control; ns indicates no significance; *p < 0.05, **p < 0.01, ***p < 0.001 vs CGN. A–L, *n* = 6.

This study used the ADR method to establish a CGN rat model (Figure 7A). Kidneys from ADR‐induced CGN rats displayed hardened texture, increased friability, and a dull surface appearance. Following treatment with PAT and DBD, the kidney coloration was significantly improved, with a light red color, soft texture restored, and surface gloss partially restored (Figure 7B). At the same time, the CGN group showed a significant decrease in body weight and a marked increase in kidney index. Following intervention with PAT and DBD, these indicators showed a marked reversal (Figure 7C,D).

To further assess the extent of kidney damage in the rats, we analyzed their urine and serum biochemical markers. In the CGN group, levels of 24‐h urine protein, SCr, and BUN were all significantly elevated. Suggesting impaired kidney function in rats, indicating successful construction of the CGN rat model. Following PAT and DBD interventions, 24‐h urine protein, SCr, and BUN levels decreased significantly (Figure 7E–H). At the same time, levels of TNF‐α and IL‐6 were elevated in the CGN group, while levels of IL‐4 and IL‐10 were reduced. Following intervention with PAT or DBD, levels of TNF‐α and IL‐6 decreased significantly, while levels of IL‐4 and IL‐10 increased significantly (Figure 7I–L). The above results indicated that DBD could effectively improve kidney function in rats, suggesting its therapeutic effect on CGN.

### 3.7 Effect of DBD on ADR‐Induced Kidney Injury in CGN Rats

**Figure 8:**
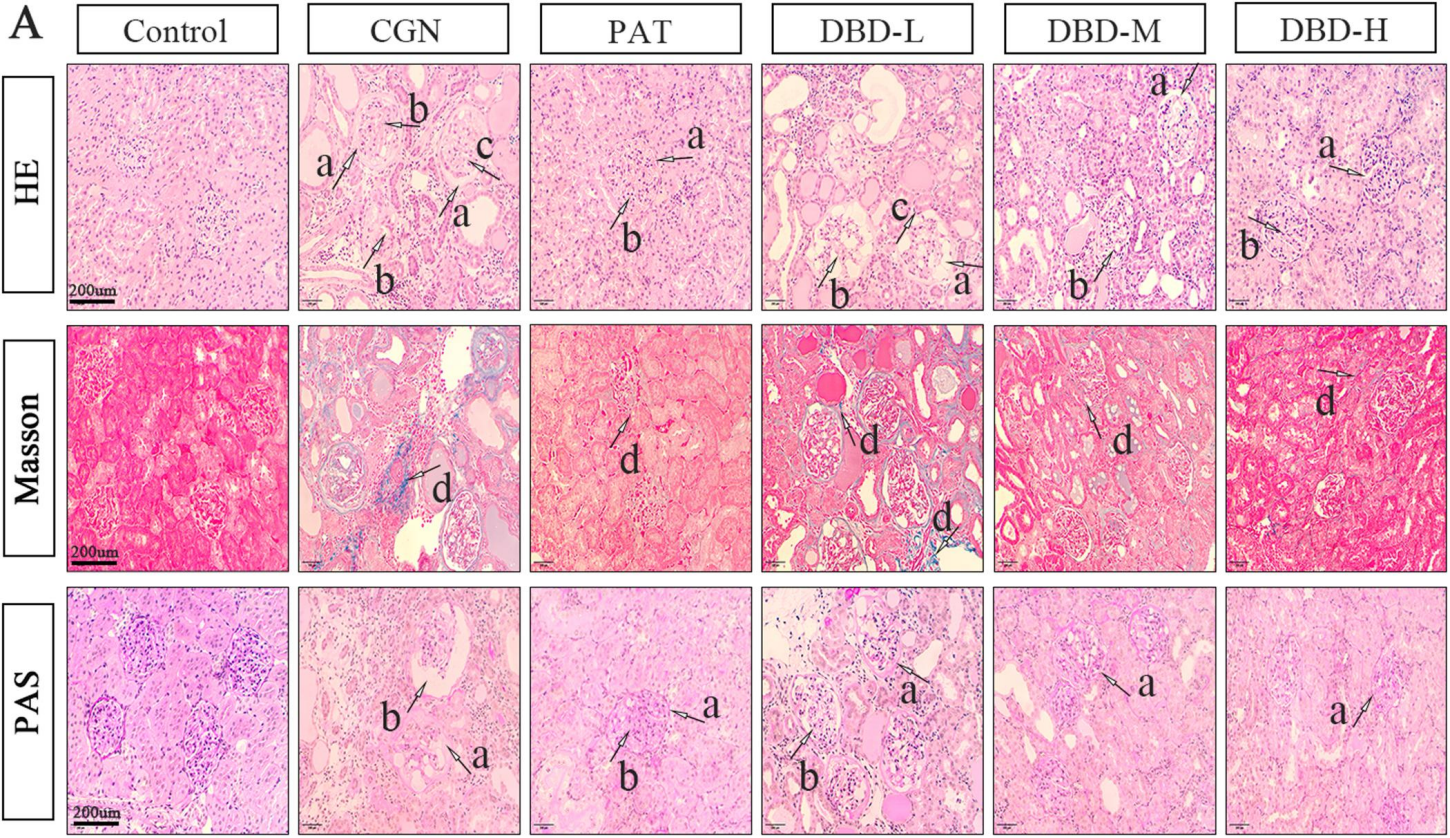
The Effect of DBD on ADR‐induced histopathological changes in the kidneys of CGN rats. (A) Representative images of HE, Masson and PAS staining of kidney tissue from each group; Pathological staining: (a) thickening of the basement membrane, (b) glomerular atrophy, (c) infiltration of inflammatory cells, (d) Matrix collagen fibers. *A*, *n* = 3.

To investigate the effect of DBD on the histopathological changes in the kidney of CGN rats, experiments were performed using HE, Masson, and PAS staining methods (Figure 8A). The CGN group exhibited significant pathological damage to the kidneys, characterized by marked enlargement of the kidneys, atrophy and deformation of the glomeruli, and thickening of the basement membrane, accompanied by interstitial collagen fiber deposition and extensive inflammatory cell infiltration. Following PAT and DBD interventions, pathological damage to the glomeruli improved significantly. Based on the above histopathological findings, it can be inferred that DBD may improve renal structural changes in CGN rats.

### 3.8 Effect of DBD on Apoptosis in Kidney Tissues of ADR‐Induced CGN Rats

**Figure 9:**
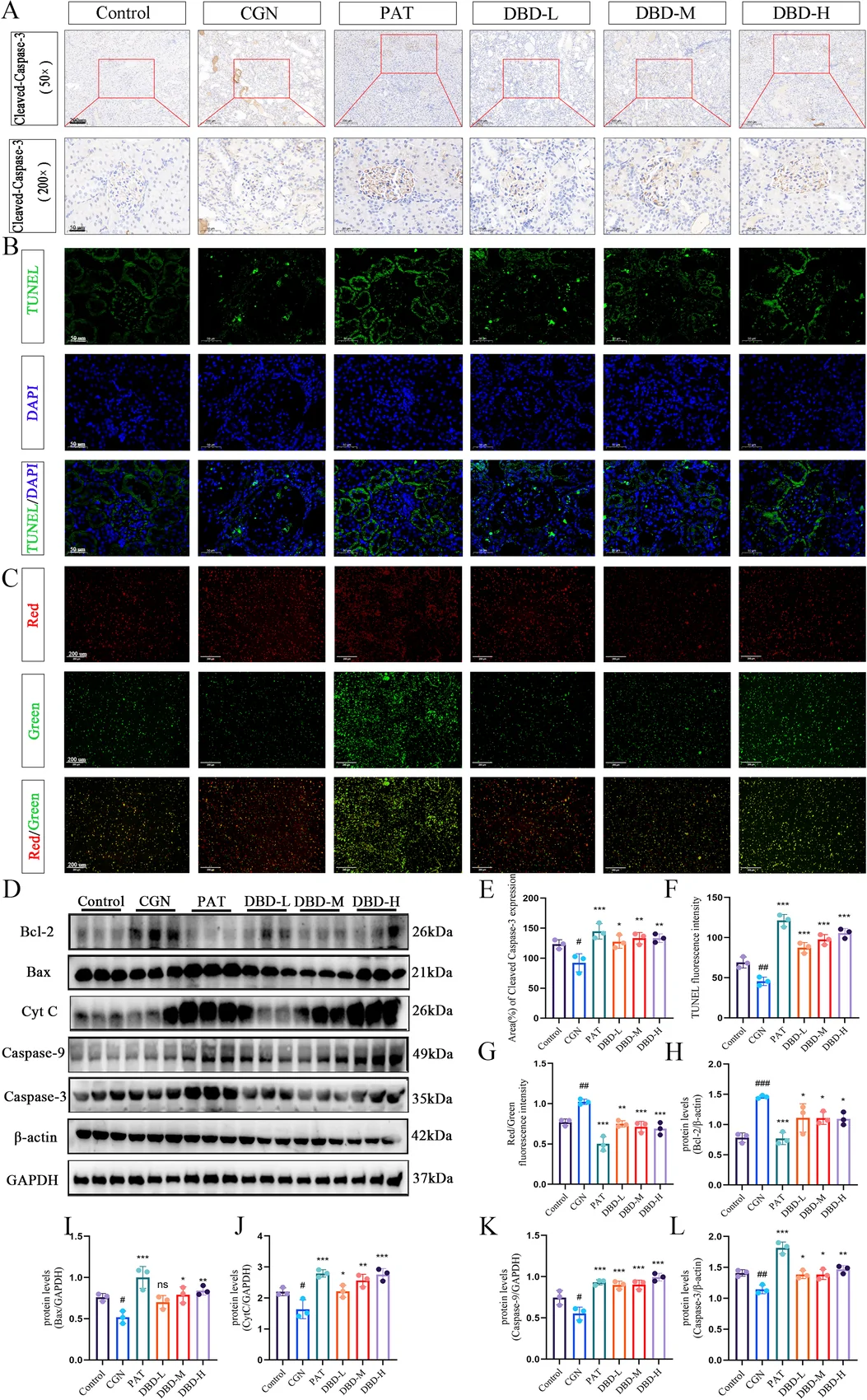
Effect of DBD on ADR‐induced apoptosis in kidney tissues of CGN rats. (A) Representative immunohistochemical images of Cleaved‐Caspase‐3 protein expression in kidney tissue; (B) TUNEL method was used to observe apoptosis in each group; (C) Intracellular MMP changes in kidney tissue in each group; (D) Protein levels of Bcl‐2, Bax, CytC, Caspase‐9, Caspase‐3, β‐actin, and GAPDH; (E) Quantitative analysis of Cleaved‐Caspase‐3 protein expression; (F) Quantitative analysis of TUNEL immunofluorescence expression; (G) visualized using the red/green ratio; (H–L) Expression of related proteins was calculated. Data are expressed as mean ± SD. ^#^ *p* < 0.05, ^##^ *p* < 0.01, ^###^ *p* < 0.001 vs Control; ns indicates no significance; *p < 0.05, **p < 0.01, ***p < 0.001 vs CGN. A–L, *n* = 3.

We used immunohistochemical assays to examine the effect of ADR on Cleaved Caspase‐3 protein levels in the kidneys of CGN rats. Studies have shown that the CGN group exhibited fewer brown‐stained areas in kidney tissue, indicating lower levels of Cleaved Caspase‐3 protein expression. However, following intervention with PAT and DBD, the brown‐stained areas increased significantly (Figure 9A).

We used the TUNEL assay to examine the effect of ADR on apoptosis in CGN rat kidney cells. The results showed that only a small number of positive cells were observed in the CGN group; however, following PAT and DBD interventions, the percentage of positive cells in rat kidney tissue increased in both groups (Figure 9B).

We evaluated the effects of DBD on MMP in ADR‐induced CGN rats using the JC‐1 staining method. The results showed that in the CGN group, mitochondrial green fluorescence was attenuated while red fluorescence was enhanced, indicating a decrease in MMP and inhibition of apoptosis in kidney tissue cells. Following intervention with PAT and DBD, these symptoms improved significantly (Figure 9C).

At the same time, the effect of DBD on apoptotic proteins in the kidney tissues of CGN rats was detected by Western Blot assay. Compared with the Control group, the protein expression levels of Bax, CytC, Caspase‐9, and Caspase‐3 in the kidney tissue of the CGN group rats were significantly downregulated, whereas the expression level of Bcl‐2 was significantly upregulated. In contrast, following treatment with PAT and DBD, the protein expression levels of Bax, CytC, Caspase‐9, and Caspase‐3 were upregulated, while the expression level of Bcl‐2 was significantly downregulated (Figure 9D). Taken together, the results of the above experiments indicate that DBD can induce apoptosis, thereby slowing the progression of CGN.

### 3.9 Effect of DBD on TLR4/NF‐κB Signaling Pathway in Kidney Tissues of Rats With ADR‐Induced CGN

**Figure 10:**
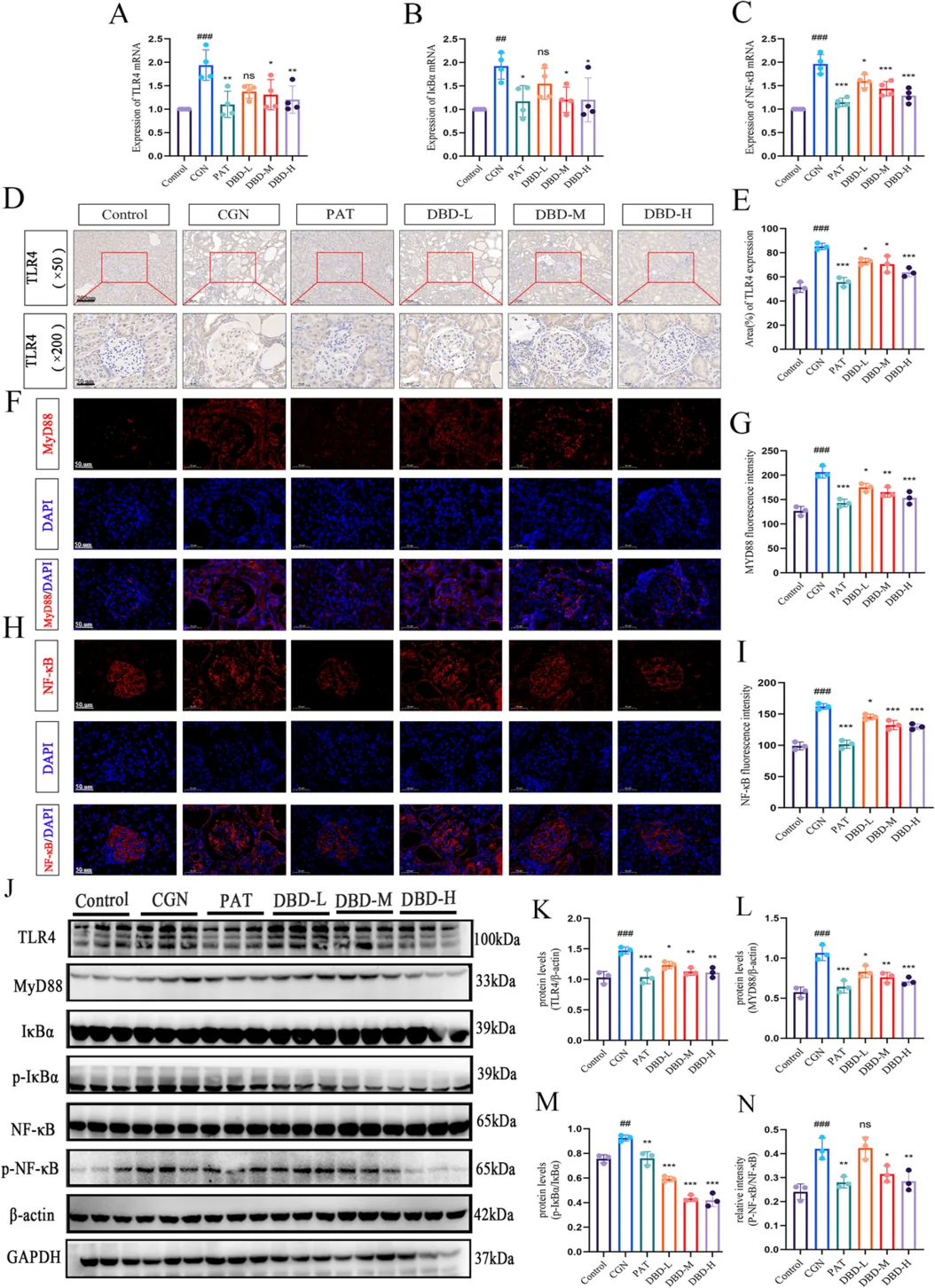
Effect of DBD on TLR4/MyD88/NF‐κB protein expression in kidney tissues of rats with ADR‐induced CGN. (A–C) Levels of mRNAs for TLR4, IκBα, and NF‐κB. (D, E) Representative images of TLR4 immunofluorescence in kidney tissue and quantitative analysis of protein expression; (F, G) Representative images of MyD88 immunofluorescence in kidney tissue and quantitative analysis of protein expression; (H, I) Representative images of NF‐κB immunofluorescence in Kidney tissue and quantitative analysis of protein expression; (J) Protein levels of TLR4, MyD88, IκBα, NF‐κB, β‐actin, and GAPDH; (K–N) expression of related proteins was calculated. Data are expressed as mean ± SD. ^#^ *p* < 0.05, ^##^ *p* < 0.01, ^###^ *p* < 0.001 vs Control; ns indicates no significance; *p < 0.05, **p < 0.01, ***p < 0.001 vs CGN. A–C, *n* = 4; D–N, *n* = 3.

To investigate the molecular mechanism of DBD in treating CGN, RT‐qPCR, Western Blot, immunohistochemistry, and immunofluorescence assays were employed to detect the expression of the TLR4/NF‐κB signaling pathway (Figure 10A–N). In the CGN group, the mRNA expression levels of TLR4, IκBα, and NF‐κB, as well as the protein expression levels of TLR4, MyD88, IκBα, and NF‐κB, were all significantly higher. Following intervention with PAT and DBD, the expression levels of these markers were significantly reduced. The above joint results suggest that DBD affects the expression of the TLR4/NF‐κB signaling pathway and ameliorates CGN disease progression.

## 4 Discussion

CGN is a diffuse or limited glomerulopathy of both kidneys, which is mainly characterized by glomerular injury, which manifests itself as chronic inflammation and fibrosis of kidney tissues, etc., which ultimately leads to the inability of the kidneys to effectively filter waste products from the blood [3]. Currently, the clinical management of CGN primarily relies on Angiotensin‐converting enzyme inhibitors to regulate blood pressure, corticosteroids, and immunosuppressants to control proteinuria, supplemented by anticoagulants to delay the progression of kidney fibrosis [19]. However, the aforementioned treatment methods often fall short of expectations due to limitations such as significant individual variations in drug sensitivity, the tendency for symptoms to recur, and long‐term medication use leading to metabolic disorders (e.g., hyperkalemia), respiratory symptoms (e.g., dry cough), increased infection risk, and adverse digestive system reactions [14]. In recent years, traditional Chinese medicine has garnered widespread attention due to its holistic regulation, multi‐targeted intervention, and relatively mild adverse reactions. In particular, the diverse bioactive components found in natural products show great promise for application in the prevention and treatment of chronic kidney disease.

Due to the long course of CGN and the deficiency of vital energy, the qi is insufficient to circulate the blood, and the blood has no qi to transport. Over time, the balance of qi and blood is disrupted, turning into blood stasis that accumulates in the kidneys. This leads to the obstruction of the qi movement within the kidneys, damage to the kidney meridians, and the loss of the essence in the kidneys, ultimately resulting in proteinuria [20]. Therefore, qi deficiency and blood stasis are the basic pathogenesis of CGN. DBD is recognized in traditional Chinese medicine theory for its efficacy in improving blood circulation and regulating metabolism [14]. ASR and AR possess anti‐inflammatory, antioxidant, and qi‐tonifying properties that fortify the body's defenses. When combined, their synergistic effects gently regulate multiple physiological functions—including hematopoiesis, immunity, and circulation—through a multi‐component interaction. Compared to Western treatments, it avoids significant side effects, demonstrates notable advantages in safety, and offers a potential strategy for combining Chinese and Western medicines to reduce toxicity and enhance efficacy. Clinically, DBD is used to treat menstrual anemia and various blood deficiency syndromes, suggesting that DBD can be used to treat CGN by tonifying Qi and generating blood [21]. However, its key active components and regulatory effects on CGN remain unclear.

Chemical composition is the material foundation for the therapeutic effect of traditional Chinese medicine. It is highly important to understand the chemical composition of compound preparations and their mechanisms of action [22]. In recent years, UPLC‐MS/MS has become a new method that is widely applied to detect natural compounds. The primary advantage of this technology lies in its high resolution and high sensitivity [23, 24, 25]. Under the background of big data, the pharmacology of traditional Chinese medicine was extensively applied in the fields of research and development, quality control, and clinical positioning of traditional Chinese medicine. Two in a combination benefits in the revealing inherent relation about TCMs multi‐way and multi‐targets characteristics and modern edge technologies. The combination of the two is conducive to exploring the intrinsic connection between the multi‐pathway and multi‐target characteristics of traditional Chinese medicine and modern cutting‐edge technologies. Therefore, in this study, we first systematically characterized the chemical constituents in DBD using UPLC‐MS/MS. And 104 major components with potential bioactivities, including Phenylpropanoids and polyketides, Lipids and lipid‐like molecules, were successfully identified by matching with relevant databases and references.

Then, by combining the above components with network pharmacological analysis, we identified 499 potential targets, among which 166 overlapping targets were expected to be associated with CGN diseases. Ten key targets, including Bcl‐2 and Caspase‐3, known to be critical in the apoptotic response, were pinpointed by CytoHubba and MCC algorithmic analyses in the PPI network. To investigate the mechanism by which DBD delays the progression of CGN, we performed an enrichment analysis using the Metscape database and found that it is significantly associated with the NF‐κB signaling pathway. To validate these results, we subsequently analyzed two independent GEO data sets to gain an initial understanding of the changes in differentially expressed genes. Since analyzing a single dataset on its own might result in the omission of differentially expressed genes due to small sample sizes or technical differences, we combined the two data sets. After merging and filtering, we ultimately obtained a list of genes that is both reliable and comprehensive. The analysis results show that this list of genes is also significantly enriched in the NF‐κB signaling pathway, which is highly consistent with the aforementioned network pharmacology findings. The results suggest that the therapeutic effects of DBD on the kidneys may be mediated by inhibition of the NF‐κB pathway. This project will provide a theoretical basis for further in‐depth research into the pathogenesis of CGN and for identifying novel therapeutic targets for CGN.

Drug‐containing serum can mitigate the interference caused by impurities and toxicity when administering crude extracts of traditional Chinese medicine in vitro. By screening for active ingredients that enter the bloodstream following in vivo metabolism in animals, it more closely reflects the actual in vivo pharmacological effects and serves as a classic method of drug administration in in vitro cellular experiments. Based on the experimental data from this study, we believe that DBD may exert its effects by regulating the TLR4/NF‐κB signaling pathway. To validate this hypothesis, we induced HBZY‐1 cells with LPS to enhance their viability [26]. The results show that the drug‐containing serum of DBD can not only inhibit the migration and invasion abilities of HBZY‐1 cells but also enhance the secretion of anti‐inflammatory factors. Finally, the preliminary observation results of the immunofluorescence experiment indicate that the drug‐containing serum of DBD can inhibit the expression of TLR4 and NF‐κB proteins. Subsequently, a CGN rat model was established using the classical ADR induction method [27]. This model is widely recognized as one of the classic models capable of effectively reproducing human glomerulonephritis [28]. As the current first‐line drug for treating CGN in clinical practice, PAT exerts its therapeutic effects through immunosuppression and anti‐inflammatory mechanisms, thereby effectively mitigating kidney damage and slowing disease progression. In this study, the extent of impaired kidney function in rats was assessed by quantitative 24‐h urine protein assay. Our study found that in rats with nephritis, kidney index, BUN, SCr, and 24‐h urine protein levels all decreased significantly following DBD intervention. Furthermore, the formation of CGN will lead to an increase in the levels of TNF‐α and IL‐6 and a decrease in the levels of IL‐10 [29, 30, 31]. This study found significantly elevated levels of TNF‐α and IL‐6 in rats with the CGN model. Previous studies have shown that DBD upregulates the anti‐inflammatory cytokines IL‐4 and IL‐10 [32, 33]. This study confirmed that, following DBD intervention, the previously decreased levels of IL‐4 and IL‐10 in the CGN group rose significantly. Consistent with previous literature, ADR can induce glomerular injury [34]. He, Masson, and PAS staining revealed reduced inflammatory cells and collagen fibers in the kidney tissues of rats in the DBD group, while simultaneously reversing glomerular atrophy and thickening of the glomerular basement membrane. These findings jointly confirmed that DBD has a definite kidney protective effect on CGN rats.

**Figure 11:**
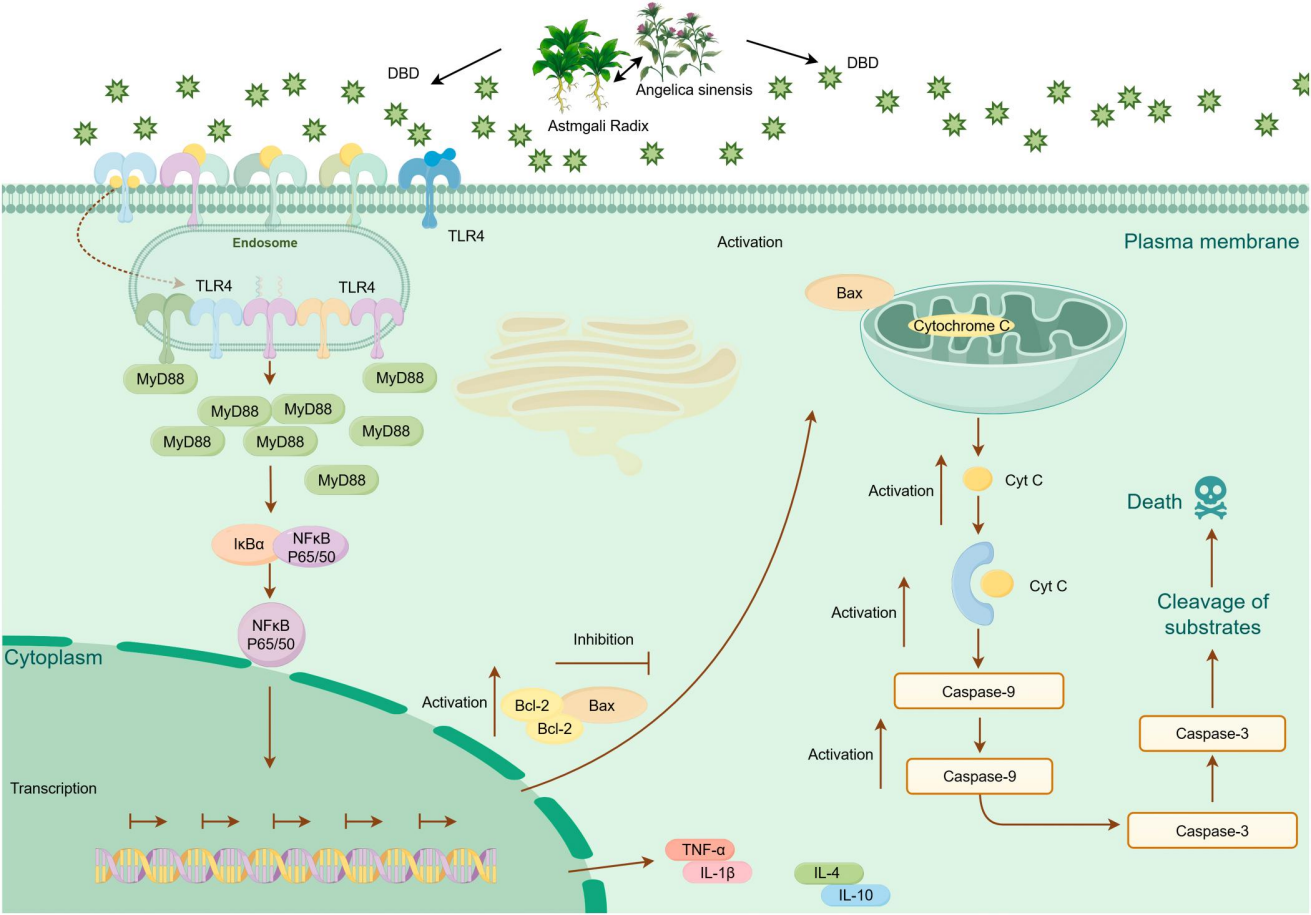
Signaling pathway diagram.

Extensive studies confirm that NF‐κB‐mediated apoptosis is critical in CGN initiation and progression [35]. LPS binds specifically to the TLR4 receptor on the surface of HBZY‐1 cells, forming a complex with MD2 through interactions such as hydrogen bonds, electrostatic forces, and hydrophobic interactions, thereby recruiting MyD88 [36, 37, 38, 39]. Thus, it can promote IκB phosphorylation degradation and initiate NF‐κB nuclear translocation [40]. Once NF‐κB enters the cell nucleus, it acts as a transcription factor, regulating the transcription of multiple genes, such as those involved in the release of TNF‐α and IL‐1β, as well as the expression of Bcl‐2, which inhibits apoptosis, thereby exacerbating the disease. When the body is subjected to ADR stimulation, it can indirectly lead to the activation of the NF‐κB signaling pathway [26, 41, 42]. When Bax is released in large quantities, it accelerates the release of CytC into the cytoplasm, which further activates Caspase‐9 and Caspase‐3, thereby activating the apoptotic pathway [43]. Apoptosis is often regarded as a double‐edged sword, playing a dual role in maintaining tissue homeostasis. The massive production of apoptotic cells poses a challenge to the local microenvironment. If apoptotic cells are not promptly and effectively cleared by macrophages, these cells may undergo secondary necrosis, releasing their contents and triggering a more intense inflammatory response and immune activation, thereby forming a vicious cycle [44]. In this study, we found through RT‐qPCR, Western blotting, immunohistochemistry, and immunofluorescence that DBD downregulated TLR4 expression, thereby reducing MyD88 protein levels, inhibiting the NF‐κB signaling pathway, and decreasing the release of inflammatory cytokines. DBD also promotes the expression of Bax and CytC proteins, which up‐regulate Caspase‐9, Caspase‐3, and Cleaved‐caspase‐3 and develops pro‐apoptotic effects (Figure 11).

## 5 Conclusion

In summary, DBD exerts its therapeutic effects on CGN by regulating the TLR4/NF‐κB signaling pathway, thereby promoting apoptosis and alleviating inflammatory responses. This result was deduced by making use of UPLC‐MS/MS technology, bioinformatics, and in vitro as well as in vivo experiments. Among them are those able to play roles, be they themselves effective components like phenylpropanoids, polyketides, lipids, and lipid‐like molecules, or organoheterocycles. In summary, this study not only provides a new perspective on elucidating the kidney protective mechanism of DBD in treating CGN but also lays a solid experimental foundation for scientifically explaining its clinical efficacy.

## Author Contributions

**Qiao Liu:** methodology, writing – original draft, software, data curation, conceptualization. **Yinghang Wang:** writing – review and editing, validation, data curation. **Xinyue Wang:** supervision, data curation, formal analysis. **Hongmei Yang:** writing – review and editing, resources. **Zhi Pan:** funding acquisition, writing – review and editing, validation, visualization. **Di Guo:** funding acquisition, writing – review and editing, investigation, project administration.

## Ethics Statement

All animal experimentation procedures were approved by the Ethics Committee of Changchun University of Chinese Medicine (Ethics account:2024799, approval date: 21 November 2024; Ethics account: 2025459, approval date:1 July 2025). This research protocol has been approved by the institutional review board of the affiliated institution and strictly adheres to local research ethics standards.

## Conflicts of Interest

The authors declare no conflicts of interest.

## Supporting information


Supporting File

## Data Availability

The data that support the findings of this study are available from the corresponding author upon reasonable request. All relevant data generated or analyzed during this study are included in this published article. Additional data are available from the corresponding author on reasonable request.
