# Supplementary material for: Exploring the Mechanism of Action of Danggui Buxue Decoction in Treating Chronic Glomerulonephritis Based on UPLC‐MS/MS and Network Pharmacology
Source: Immun Inflamm Dis. 2026 Sep 11;14(9):e70497. doi: 10.1002/iid3.70497 (PMC13564246; doi:10.1002/iid3.70497)
Supplement: Supplementary file 1 [file IID3-14-e70497-s001.docx]

**Exploring the Mechanism of action of Danggui Buxue Decoction in Treating Chronic Glomerulonephritis Based on UPLC-MS/MS and Network Pharmacology**

**Qiao Liu^1†^, Yinghang Wang^2†^, Xinyue Wang^1^, Hongmei Yang^4^, Zhi Pan^1*^, Di Guo^3*^**

**^1^****Fangzheng Research Laboratory, Jilin Ginseng Academy, Changchun University of Chinese Medicine,Changchun,China,15143796326@163.com,1986218439@qq.com,panzhi@ccucm.edu.cn,^2^College of Traditional Chinese Medicine, Changchun University of Chinese Medicine, Changchun, China,** **panzhiwyh@sohu.com, ^3^****College of Basic Medicine, Changchun University of Chinese Medicine, Changchun, China,** **guo_di2023@163.com.** **^4^Public Experimental Center, Changchun University of Chinese Medicine, Changchun, China, yanghm0327@sina.cn.**

***Correspondence:
Zhi Pan**
panzhi@ccucm.edu.cn

**Di Guo**
guo_di2023@163.com

**^†^**These authors share first authorship

Table S1

Detailed Information on the Reagents

| **Reagent** | **Catalog**  **number** | **Dilution** | **Company** | **Area** |
| --- | --- | --- | --- | --- |
| PAT | d2408181 |  | Xinxiang Changle Pharmaceutical Co., Ltd. | China |
| ADR | H14023143 |  | Shanxi Powerdone Pharmaceutics Co., Ltd | China |
| TNF-α (pg・mL-1) | YX-201407R |  | Shanghai Youxuan Biotechnology Co., Ltd | China |
| IL-6 (pg・mL-1) | YX-091206R |  | Shanghai Youxuan Biotechnology Co., Ltd | China |
| IL-4 (pg・mL-1) | YX-21186R |  | Shanghai Youxuan Biotechnology Co., Ltd | China |
| IL-10 (pg・mL-1) | YX-21190R |  | Shanghai Youxuan Biotechnology Co., Ltd | China |
| SCr | C011-2-1 |  | Nanjing Chengjian Institute of Biotechnology | China |
| 24 hour urine protein | C035-2-1 |  | Nanjing Chengjian Institute of Biotechnology | China |
| BUN | C013-2-1 |  | Nanjing Chengjian Institute of Biotechnology | China |
| HE | G1076 |  | Servicebio | China |
| Masson | G1006 |  | Servicebio | China |
| PAS | G1008 |  | Servicebio | China |
| Mitochondrial Extraction Kit | C3606 |  | Beyotime Biotechnology | China |
| JC-1 Test Kit | C2003S |  | Beyotime Biotechnology | China |
| TLR4 | 19811-1-AP | 1:1000 | Proteintech Group | China |
| MyD88 | GB111554-100 | 1:1000 | Servicebio | China |
| Cleaved-Caspase-3 | GB11532-100 | 1:1000 | Servicebio | China |
| IκBα | A19714 | 1:1000 | Abclonal | USA |
| P-IκBα | AP0707 | 1:1000 | Abclonal | USA |
| NF-κB | A19653 | 1:1000 | Abclonal | USA |
| P-NF-κB | AP1355 | 1:1000 | Abclonal | USA |
| Bax | A19684 | 1:1000 | Abclonal | USA |
| CytC | A1561 | 1:1000 | Abclonal | USA |
| Caspase-9 | A18676 | 1:1000 | Abclonal | USA |
| Caspase-3 | A110211 | 1:1000 | Abclonal | USA |
| Bcl-2 | A19693 |  | Abclonal |  |

Table S2

Detailed Information on Components Identified by UPLC-MS/MS.

| **No.** | **Compound** | **RT** | **Measured** | **Adduct ion** | **Formula** | **Delta** | **MS^2^ ion** | **Source** |
| --- | --- | --- | --- | --- | --- | --- | --- | --- |
|  |  | **(min)** | **(m/z)** |  |  | **(ppm)** | **(m/z)** |  |
| 1 | 4-Hydroxybenzoic acid | 6.3511 | 137.0243 | M-H | C7H6O3 | -0.9 | 93.0344,109.0288 | (Lv et al., 2024) |
| 2 | L-Glutamic acid | 0.7650 | 146.0458 | M-H | C5H9NO4 | -0.4 | 128.0350,102.0558 | (Tong et al., 2024) |
| 3 | Calycosin | 6.0084 | 283.0613 | M-H | C16H12O5 | 0.4 | 268.0375,239.0349,211.0402,135.0084 | (Wang et al., 2019) |
| 4 | Formononetin | 8.8588 | 267.066 | M+Cl, 2M-H, M-H | C16H12O4 | -1.0 | 252.0426,251.0348,223.0400,195.0451,132.0213 | (Fang et al., 2023) |
| 5 | Sucrose | 0.8203 | 387.1155 | M+HCOO | C12H22O11 | 3.3 | 341.1091,59.0137 | NA |
| 6 | Orsellinic acid | 4.9536 | 167.0349 | M-H | C8H8O4 | -0.3 | 123.0449,152.0111 | NA |
| 7 | Caffeic acid | 5.3958 | 179.0351 | M-H | C9H8O4 | 0.7 | 135.0449, 134.0373,107.0499 | (Lv et al., 2024) |
| 8 | Cryptochlorogenic acid | 4.9124 | 353.0886 | M-H | C16H18O9 | 2.2 | 173.0458,191.0560,179.0351 | (Z. T. Zhang et al., 2024) |
| 9 | Isomucronulatol 7-O-glucoside | 6.2952 | 463.1627 | M-H | C23H28O10 | 3.7 | 301.1079, 271.0604,164.0473,135.0451,121.0292 | (Li et al., 2019) |
| 10 | L-Histidine | 0.7290 | 154.0623 | M-H | C6H9N3O2 | 0.3 | 93.0457,110.0722,137.0353 | (Gong et al., 2025) |
| 11 | Lucidal | 12.3552 | 471.3499 | M-H+H2O | C30H46O3 | 5.5 | 199.1416,407.3318,425.3425,453.3323 | NA |
| 12 | Medicarpin | 9.4509 | 269.0821 | M-H | C16H14O4 | 0.8 | 254.0579 | (Lin et al., 2024) |
| 13 | Citric acid | 0.9645 | 191.0198 | M-H | C6H8O7 | 0.3 | 111.0084,87.0085,173.0089 | (Z. T. Zhang et al., 2024) |
| 14 | Vanillin | 6.1628 | 151.0401 | M-H | C8H8O3 | 0.1 | 136.0164,59.0137 | (J. Zhang et al., 2024) |
| 15 | Sinapic acid | 6.6849 | 223.0616 | M-H | C11H12O5 | 1.8 | 208.0376,164.0477,149.0242,121.0291,93.0345 | (Yang et al., 2023) |
| 16 | Hydroxygenkwanin | 8.4320 | 299.0566 | M-H | C16H12O6 | 1.5 | 284.0325,256.0366 | (J. Zhang et al., 2024) |
| 17 | Methyl hexadecanoate | 10.8903 | 315.2551 | M+HCOO | C17H34O2 | 3.6 | 315.2544,313.2380,297.2438,127.1122 | (J. Zhang et al., 2024) |
| 18 | Gallic acid | 1.6143 | 169.0144 | M-H | C7H6O5 | 1.1 | 125.0241,97.0293 | (Fang et al., 2023) |
| 19 | Protocatechuic acid | 4.9124 | 153.0194 | M-H | C7H6O4 | 0.3 | 109.0292 | (Lv et al., 2024) |
| 20 | Diosmetin-7-O-Beta-D-glucopyranoside | 5.5940 | 461.1103 | M-H | C22H22O11 | 2.9 | 299.0564,284.0326 | NA |
| 21 | Azelaic acid | 6.8078 | 187.0976 | M-H, 2M-H, M+Cl | C9H16O4 | 0.2 | 125.097,123.0810 | (Z. T. Zhang et al., 2024) |
| 22 | Sophoricoside | 6.5103 | 431.1003 | M-H | C21H20O10 | 4.5 | 268.0373,240.0418 | (Wang et al., 2019) |
| 23 | Isoliquiritigenin | 7.4740 | 255.0663 | M-H | C15H12O4 | 0.3 | 135.0086,119.0500,91.0189 | (Wang et al., 2019) |
| 24 | Prunetin | 9.8171 | 283.0616 | M-H | C16H12O5 | 1.4 | 268.0367,240.0417 | NA |
| 25 | Glycitein | 7.6069 | 283.061 | M+Cl, 2M-H, M-H | C16H12O5 | -0.8 | 239.0348,268.0377 | (Zhu et al., 2024) |
| 26 | 6-Gingerol | 9.8326 | 293.1763 | M-H | C17H26O4 | 1.6 | 221.1544,177.0916 | (Lin et al., 2024) |
| 27 | Pectolinarigenin | 6.4204 | 313.0723 | M-H | C17H14O6 | 1.9 | 298.0484,270.0530 | (Lin et al., 2024) |
| 28 | Stearidonic Acid | 10.1214 | 293.2126 | M-H+H2O | C18H28O2 | 3.6 | 275.2032,249.2258,113.0970,125.0973 | NA |
| 29 | 3-Hexen-1-ol O-b-D-glucopyranoside | 6.4865 | 261.1346 | M-H | C12H22O6 | 0.8 | 125.0969,187.0975 | (J. Zhang et al., 2024) |
| 30 | Gentisic acid | 3.5608 | 153.0194 | M-H | C7H6O4 | 0.7 | 109.0292,91.0189 | (Lin et al., 2024) |
| 31 | 2-Hydroxy-1,8-cineole | 8.1183 | 215.129 | M+HCOO | C10H18O2 | 0.9 | 197.1182,153.1283 | NA |
| 32 | Uridine diphosphate glucose | 0.9645 | 611.0481 | M+HCOO | C15H24N2O17P2 | -9.1 | 111.0198,78.9589 | NA |
| 33 | Genistein | 6.6451 | 269.0459 | M-H | C15H10O5 | 1.2 | 133.0293,181.0647 | (Lin et al., 2024) |
| 34 | Plumieride | 3.2168 | 515.1424 | M+HCOO | C21H26O12 | 3.8 | 179.0349,135.0450 | NA |
| 35 | Ascotrichic acid | 9.9471 | 269.176 | M-H | C15H26O4 | 0.6 | 207.1755 | NA |
| 36 | Guanosine | 1.4136 | 282.0846 | M-H, 2M-H | C10H13N5O5 | 0.8 | 150.042,133.0155 | (Lin et al., 2024) |
| 37 | gamma-Linolenic acid | 8.6222 | 295.2283 | M-H+H2O | C18H30O2 | 3.7 | 277.2172,195.1387,171.1024 | NA |
| 38 | 2'-O-Methyladenosine | 1.1209 | 326.1112 | M+HCOO | C11H15N5O4 | 2.2 | 134.047 | NA |
| 39 | Apigenin 6,8-di-C-alpha-L-arabinopyranoside | 5.4623 | 551.1411 | M-H+H2O | C25H26O13 | 2.0 | 137.0242,93.0344 | NA |
| 40 | 2-Carboxybenzaldehyde | 5.6758 | 149.0245 | M-H | C8H6O3 | 0.4 | 121.0292,77.0395,93.0342,105.0339 | NA |
| 41 | Swertiamarin | 2.3329 | 433.1372 | M+CH3COO | C16H22O10 | 5.6 | 301.0925, 138.0320,124.0162,123.0084,71.0137,59.0136 | NA |
| 42 | Liquiritin | 6.1109 | 417.1202 | M-H | C21H22O9 | 2.6 | 255.0099,135.0086,119.0500,91.0189 | (Zhu et al., 2024) |
| 43 | Hexylitaconic acid | 6.8801 | 231.124 | M-H+H2O | C11H18O4 | 3.9 | 185.1184,141.1286 | NA |
| 44 | Hanphillin | 7.6643 | 265.1448 | M-H+H2O | C15H20O3 | 3.3 | 247.1340,237.1485,203.1436,205.1231,95.0502 | NA |
| 45 | Pantoyllactone glucoside | 0.9500 | 383.1204 | M+HCOOH+HCOO | C12H20O8 | 3.0 | 341.1096,323.0986,59.0137,71.0137,73.0294,101.0242,113.0241 | NA |
| 46 | Emodin anthrone | 8.1938 | 273.0769 | M-H+H2O | C15H12O4 | 2.3 | 272.0594,259.0585,258.0533,136.0163,135.0084,122.0372,108.0214,109.0292,91.0189 | NA |
| 47 | Isoboonein | 8.5112 | 169.0871 | M-H | C9H14O3 | 0.4 | 125.0969,123.0820,71.0502 | NA |
| 48 | Scillarenin | 9.5167 | 427.2139 | M+HCOO | C24H30O4 | 3.4 | 399.1818,355.1919,255.1755 | NA |
| 49 | Strophanthidin (Corchorin) | 5.7567 | 449.2157 | M+HCOO | C23H32O6 | -5.9 | 359.1829,287.1613,73.0294 | NA |
| 50 | p-Coumaric acid | 6.0614 | 163.0401 | M-H | C9H8O3 | 0.3 | 119.0499 | (Z. T. Zhang et al., 2024) |
| 51 | L-Tryptophan | 3.7622 | 203.0827 | M-H, 2M-H | C11H12N2O2 | 0.4 | 116.0505,142.0663,74.0247 | (Wang et al., 2019) |
| 52 | Dichlorisone Acetate | 1.1100 | 485.1522 | M-H+CH3OH | C23H28Cl2O5 | 5.3 | 341.1097,57.0345,59.0137,71.0137,89.0243,101.0244,142.0508 | NA |
| 53 | ADENOSINE | 1.1180 | 268.1035 | M+H | C10H13N5O4 | -2.1 | 136.0614,119.0348 | (Jiang et al., 2022) |
| 54 | Ononin | 7.0278 | 431.1326 | M+H | C22H22O9 | -2.4 | 269.0801,254.0562,213.0911 | (Fang et al., 2023) |
| 55 | Calycosin-7-O-Beta-D-glucoside | 6.0289 | 447.1275 | M+H, 2M+H | C22H22O10 | -2.4 | 270.0518,253.0490 | (Li et al., 2019) |
| 56 | L-PROLINE | 0.9004 | 116.0706 | M+H | C5H9NO2 | -0.3 | 70.0656,68.0499 | (Z. T. Zhang et al., 2024) |
| 57 | Cordycepin | 1.8625 | 252.1086 | M+H | C10H13N5O3 | -2.0 | 136.0615,99.0439 | (J. Zhang et al., 2024) |
| 58 | Biochanin A | 9.8128 | 285.0751 | M+H | C16H12O5 | -2.1 | 270.0518,213.0543 | (Z. T. Zhang et al., 2024) |
| 59 | Ligustilide | 12.5979 | 191.1063 | M+H | C12H14O2 | -1.7 | 173.0958,163.1110,145.1010,117.0699 | (Wei & Huang, 2015) |
| 60 | Guanine | 1.4150 | 152.0564 | M+H | C5H5N5O | -1.6 | 110.0349,135.0298 | (Wang et al., 2021) |
| 61 | Physalin A | 5.2595 | 581.1972 | M+CH3OH+Na | C28H30O10 | -5.2 | 229.0873,107.0496 | NA |
| 62 | Retrochalcone | 8.0847 | 271.0959 | M+H | C16H14O4 | -2.1 | 229.0855,177.0543,121.0278,107.0492,123.0439 | NA |
| 63 | ADENINE | 1.7598 | 136.0615 | M+H | C5H5N5 | -2.0 | 119.0349,94.0403 | (Z. T. Zhang et al., 2024) |
| 64 | Cyclocarioside J | 9.1712 | 605.4044 | M+H-H2O | C35H58O9 | -0.6 | 143.1058,125.0963,121.1027,107.0858 | NA |
| 65 | Geldanamycin | 5.6181 | 525.2646 | M+H-2H2O | C29H40N2O9 | 9.1 | 507.2545,489.2426,441.2213,200.0914, | NA |
| 66 | Homoplantaginin | 6.5907 | 463.1228 | M+H | C22H22O11 | -1.4 | 301.0700,286.0466 | (Lin et al., 2024) |
| 67 | Astilbin | 6.2727 | 433.1125 | M+H-H2O, M+2CH3CN+H | C21H22O11 | -1.0 | 271.0593 | NA |
| 68 | Propionic acid | 7.2790 | 74.06049 | M+NH4-H2O | C3H6O2 | -1.3 | 56.0500,57.0340 | NA |
| 69 | 4-Methoxysalicylic acid | 5.2704 | 169.0493 | M+H | C8H8O4 | -1.2 | 151.0386,95.0857 | (Lin et al., 2024) |
| 70 | L-Threonine | 0.7951 | 120.0655 | M+H | C4H9NO3 | 0.0 | 74.0604,56.0500 | (Gong et al., 2025) |
| 71 | Cytosine | 0.9178 | 112.0506 | M+H | C4H5N3O | 0.8 | 94.0402,95.0242 | (Gong et al., 2025) |
| 72 | Enniatin B | 13.3832 | 657.4429 | M+NH4 | C33H57N3O9 | -0.7 | 86.0967,186.1485,196.1329,214.1433 | NA |
| 73 | Levistilide A | 11.5456 | 399.216 | M+H2O+H | C24H28O4 | -3.1 | 191.1062,173.0960,145.1007,381.2056 | NA |
| 74 | Arjunic acid | 8.3990 | 507.3676 | M+H2O+H | C30H48O5 | -2.0 | 489.3573,277.2167,81.0702,67.0547,55.0548,79.0547 | NA |
| 75 | Sec-O-glucosylhamaudol | 7.0566 | 461.1434 | M+Na | C21H26O10 | 3.7 | 299.0906 | NA |
| 76 | Obscuraminol D | 9.4352 | 288.2891 | M+CH3OH+H | C16H33NO | -2.5 | 106.0863,85.1014,88.0759,57.0704 | NA |
| 77 | Podophyllotoxin 4-O-glucoside | 7.5521 | 594.2174 | M+NH4 | C28H32O13 | -1.2 | 301.1071,167.0699 | NA |
| 78 | Alprostadil | 8.4125 | 354.2637 | M+NH4-H2O | C20H34O5 | -2.2 | 353.2288,163.1108,177.1271 | NA |
| 79 | Ferulic acid | 6.3371 | 195.065 | M+H-H2O, M+H | C10H10O4 | -1.1 | 145.0281,177.0543,149.0593 | (Z. T. Zhang et al., 2024) |
| 80 | Coleonol B | 5.2704 | 451.2286 | M+H2O+Na | C22H34O7 | -5.2 | 56.0501,4332179 | NA |
| 81 | 1-Linoleoyl-sn-glycero-3-phosphorylcholine | 11.1167 | 520.3394 | M+H | C26H50NO7P | -0.6 | 502.3279,184.0729,104.1070,86.0967,60.0813 | (J. Zhang et al., 2024) |
| 82 | Nicotinamide | 1.4548 | 123.0552 | M+H | C6H6N2O | -0.5 | 96.0441,78.0342,80.0499,106.0287 | (Zhu et al., 2024) |
| 83 | Apigenin 7-O-glucuronide | 6.6517 | 519.113 | M+CH3OH+Na+H2O | C21H18O11 | 3.5 | 271.0592 | NA |
| 84 | p-Hydroxy-5,6-dehydrokawain | 6.0289 | 285.0751 | M+H2O+Na | C14H12O4 | 5.0 | 270.0517,269.0438,225.0542,214.0621,213.0543,137.0231,134.0361 | NA |
| 85 | Alpha-Linolenic acid | 11.6818 | 296.2578 | M+NH4 | C18H30O2 | -2.1 | 279.231,95.0858 | NA |
| 86 | Semialactone | 11.5612 | 467.315 | M+H-H2O | C30H44O5 | -1.2 | 449.3038,379.2628,93.0700 | NA |
| 87 | 6-O-Benzoylphlorigidoside B | 6.8821 | 573.1594 | M+Na-H2O | C26H32O14 | 2.7 | 285.0747,270.0507, | NA |
| 88 | Nelumnucifoside B | 5.3277 | 453.244 | M+CH3OH+Na | C21H34O7 | -6.0 | 435.2326,69.0339 | NA |
| 89 | 3'-Hydroxyxanthyletin | 8.0033 | 299.0908 | M+CH3OH+Na | C14H12O4 | 5.2 | 284.0679,283.0596,238.0621,239.0703,255.0639,266.0569,210.0673,196.0519 | NA |
| 90 | Chromomoric acid B | 9.8822 | 311.221 | M+H2O+H | C18H28O3 | -4.1 | 293.2104,275.1995,163.1114,55.0548,81.0701,83.0858 | NA |
| 91 | Ganoderiol A | 8.6984 | 457.3673 | M+H-H2O | C30H50O4 | -0.6 | 439.3574,421.3452,81.0702,107.0856,119.0854,121.1010 | NA |
| 92 | L-Phenylalanine | 5.6928 | 166.086 | M+H | C9H11NO2 | -1.3 | 103.0543,120.0806 | (Yang et al., 2023) |
| 93 | Marmesin | 8.4332 | 247.0961 | M+H | C14H14O4 | -1.5 | 229.0847,175.0389 | (Lin et al., 2024) |
| 94 | Senkyunolide I | 7.8235 | 223.0979 | M-H | C12H16O4 | 1.4 | 207.1028,181.1234,179.1070,161.0972,163.1129,95.0503,59.0138 | (J. Zhang et al., 2024) |
| 95 | Senkyunolide G | 9.0406 | 207.1027 | M-H | C12H16O3 | 0.2 | 161.0969,163.1126,206.0873,83.0501,85.0655 | NA |
| 96 | Senkyunolide C | 7.3401 | 249.0769 | M+HCOO | C12H12O3 | 0.4 | 203.0713,205.0870,189.0570,174.0323,175.0401,121.0290,83.0501,59.0137 | NA |
| 97 | Isoastragaloside I | 10.7053 | 869.4888 | M+H-H2O, M+H | C45H72O16 | -0.6 | 157.0496,143.1067,97.0286 | (Tong et al., 2024) |
| 98 | Senkyunolide S | 7.2634 | 223.0961 | M+H-H2O | C12H16O5 | -1.7 | 149.0594,177.0907 | NA |
| 99 | Senkyunolide A | 8.5089 | 211.1325 | M+H2O+H | C12H16O2 | -4.6 | 193.1215,147.1164,119.0854,105.0699,91.0545,81.0702,79.0546,67.0546,55.0547 | NA |
| 100 | Astragaloside III | 8.5460 | 767.4567 | M+H-H2O, M+NH4 | C41H68O14 | -1.2 | 455.3517,437.3400,143.1064,141.1270,125.0961,109.1013 | NA |
| 101 | Astragaloside IV | 7.8548 | 785.467 | M+H | C41H68O14 | -1.6 | 143.1064,437.3404 | (Tong et al., 2024) |
| 102 | Methylnissolin-3-O-glucoside | 7.2886 | 521.1677 | M+HCOO, M+CH3COO, M+Cl, M-H | C23H26O10 | 2.8 | 299.0927,284.0691,269.0452 | NA |
| 103 | 3-Butylidenephthalide | 8.9469 | 189.0906 | M+H, M+H2O+H, M+H-H2O | C12H12O2 | -2.0 | 128.0619,133.0281 | NA |
| 104 | Soyasaponin Bb | 8.7904 | 943.5252 | M+H | C48H78O18 | -0.9 | 441.3719,423.3615 | (Z. T. Zhang et al., 2024) |

NA: relevant references were not identified in the literature search

**References**

Fang, C., Zhang, J., Han, J., Lei, Y., Cao, Z., Pan, J., Pan, Z., Zhang, Z., Qu, N., Luo, H., Ma, Y., Han, D, 2023. Tiaogan Jiejiu Tongluo Formula attenuated alcohol-induced chronic liver injury by regulating lipid metabolism in rats. J Ethnopharmacol. 317, 116838. <https://doi.org/10.1016/j.jep.2023.116838>

Gong, K., Zhou, S., Xiao, L., Xu, M., Zhou, Y., Lu, K., Yu, X., Zhu, J., Liu, C., Zhu, Q, 2025. Danggui Shaoyao San ameliorates Alzheimer's disease by regulating lipid metabolism and inhibiting neuronal ferroptosis through the AMPK/Sp1/ACSL4 signaling pathway. Front Pharmacol. 16, 1588375. <https://doi.org/10.3389/fphar.2025.1588375>

Jiang, M., Cao, J., Zhang, C., Su, B., Wang, S., Ning, N., Lei, T., Li, P, 2022. A comprehensive strategy for quality evaluation of Wushe Zhiyang Pills by integrating UPLC-DAD fingerprint and multi-ingredients rapid quantitation with UPLC-MS/MS technology. J Pharm Biomed Anal. 210, 114556. <https://doi.org/10.1016/j.jpba.2021.114556>

Li, Y., Guo, S., Zhu, Y., Yan, H., Qian, D. W., Wang, H. Q., Yu, J. Q., Duan, J. A, 2019. Comparative analysis of twenty-five compounds in different parts of Astragalus membranaceus var. mongholicus and Astragalus membranaceus by UPLC-MS/MS. J Pharm Anal. 9(6), 392-399. <https://doi.org/10.1016/j.jpha.2019.06.002>

Lin, X., Lin, J., Ji, L., Zhang, J., Zhang, Y., Hong, J., Li, G., Lin, X, 2024. Protective effect of Haoqin Qingdan decoction on pulmonary and intestinal injury in mice with influenza viral pneumonia. Front Pharmacol. 15, 1449322. <https://doi.org/10.3389/fphar.2024.1449322>

Lv, Z., Li, C., Wu, T., Zhao, P., Liu, Y., Ouyang, H., Feng, J., He, J, 2024. Development of a high sensitivity UHPLC-MS/MS method to determine the twelve compounds of Physochlainae Radix extract and application to a pharmacokinetic study in rats. Arabian Journal of Chemistry. 17(4).

Tong, Q., Chang, Y., Shang, G., Yin, J., Zhou, X., Wang, S., Yan, X., Zhang, F., Wang, S., Yao, W, 2024. Integrated chemical characterization, metabolite profiling, and pharmacokinetics analysis of Zhijun Tangshen Decoction by UPLC-Q/TOF-MS. Front Pharmacol. 15, 1363678. <https://doi.org/10.3389/fphar.2024.1363678>

Wang, D., Liu, Y., Tang, D., Wei, S., Sun, J., Ruan, L., He, L., Li, R., Ren, Q., Tian, X., Chen, Y, 2021. Induction of PI3K/Akt-Mediated Apoptosis in Osteoclasts Is a Key Approach for Buxue Tongluo Pills to Treat Osteonecrosis of the Femoral Head. Front Pharmacol. 12, 729909. <https://doi.org/10.3389/fphar.2021.729909>

Wang, Y., Liu, L., Ma, Y., Guo, L., Sun, Y., Liu, Q., Liu, J, 2019. Chemical Discrimination of Astragalus mongholicus and Astragalus membranaceus Based on Metabolomics Using UHPLC-ESI-Q-TOF-MS/MS Approach. Molecules. 24(22). <https://doi.org/10.3390/molecules24224064>

Wei, W. L., Huang, L. F, 2015. Simultaneous determination of ferulic acid and phthalides of Angelica sinensis based on UPLC-Q-TOF/MS. Molecules. 20(3), 4681-4694. <https://doi.org/10.3390/molecules20034681>

Yang, R., Wei, L., Wang, J., Huang, S., Mo, P., Chen, Q., Zheng, P., Chen, J., Zhang, S., Chen, J, 2023. Chemical characterization and metabolic profiling of Xiao-Er-An-Shen Decoction by UPLC-QTOF/MS. Front Pharmacol. 14, 1219866. <https://doi.org/10.3389/fphar.2023.1219866>

Zhang, J., Shen, W., Liu, F., He, H., Han, S., Luo, L, 2024. Integrated approach with UHPLC-Q-Exactive-tandem mass spectrometry, network analysis, and molecular docking to determine potential active compounds and mechanisms of Rhizoma Musae decoction in osteoarthritis treatment. Front Pharmacol. 15, 1380335. <https://doi.org/10.3389/fphar.2024.1380335>

Zhang, Z. T., Qi, Y., Chen, P., Chen, L., Jiang, Y., Fan, Z., Guan, H., Bai, L., Liu, J., Zhao, D., Yan, G, 2024. Dang-Gui-Bu-Xue decoction against diabetic nephropathy via modulating the carbonyl compounds metabolic profile and AGEs/RAGE pathway. Phytomedicine. 135, 156104. <https://doi.org/10.1016/j.phymed.2024.156104>

Zhu, X., Lin, M., Chi, Y., Li, X., Jiang, Z., Jian, X., Lian, M., Wu, X., Han, S., Shi, X, 2024. Qualitative and quantitative analysis of chemical components in Qianggan capsule by UHPLC-Q-TOF-MS/MS and LC-sMRM. J Chromatogr A. 1728, 465020. <https://doi.org/10.1016/j.chroma.2024.465020>
